# Luteolin and Naringenin from Herbal Medicines Suppress Pulmonary Arterial Smooth Muscle Cell Hyperproliferation in Pulmonary Arterial Hypertension by Modulating IGF1R/PI3K/AKT Signaling

**DOI:** 10.3390/ijms272110018

**Published:** 2026-11-08

**Authors:** Hanghang Gou, Liping Chen, Chengxun He, Qi Liang, Ke Liu, Yi Zhu, Xiuli Yang, Wei Peng

**Affiliations:** 1 Chinese Medicine Germplasm Resources Innovation and Effective Uses Key Laboratory of Sichuan Province, School of Modern Chinese Medicine Industry, School of Pharmacy, Chengdu University of Traditional Chinese Medicine, Chengdu 611137, China; ghh@stu.cdutcm.edu.cn (H.G.); chenliping@stu.cdutcm.edu.cn (L.C.); liangqi@stu.cdutcm.edu.cn (Q.L.); 2 Department of Respiratory Medicine, Hospital of Chengdu University of Traditional Chinese Medicine, Chengdu 610032, China; hechengxun@stu.cdutcm.edu.cn (C.H.); liuke@stu.cdutcm.edu.cn (K.L.); zhytcm188@stu.cdutcm.edu.cn (Y.Z.); yangxiuli@keele.cdutcm.edu.cn (X.Y.)

**Keywords:** pulmonary arterial hypertension, natural products, multi-omics, bioinformatics analysis, IGF1R/PI3K/AKT

## Abstract

Pulmonary arterial hypertension (PAH) remains a progressive disease with limited therapies. As part of our continuing study to identify effective agents for PAH from natural herbal medicines, we analyzed the chemical constituents of an empirically validated herbal formula named *Guilou Tongluo* formula (GLTL) by UHPLC-Q-Exactive MS, retrieved its putative blood-absorbed constituents from databases, and integrated them with lung RNA-sequencing data to discover potential active constituents against PAH. A compound–disease network was constructed; drug–disease intersection genes underwent functional enrichment analysis, whereas hub targets were prioritized by protein–protein interaction analysis. Immune infiltration and single-cell RNA sequencing were used to analyze the vascular microenvironment, while nomogram and decision curve analyses exploratorily assessed the association of candidate targets with disease status at the tissue level. Target engagement was predicted by molecular docking and molecular dynamics simulation and supported by cellular thermal shift and drug affinity target stability assays; the mechanism of the constituents was then examined. The results showed that GLTL attenuated proliferation and promoted apoptosis in PDGF-BB-stimulated pulmonary arterial smooth muscle cells (PASMCs), an effect associated with IGF1R/PI3K/AKT signaling in smooth muscle cells, with IGF1R identified as a key candidate target. Molecular docking and dynamics simulation predicted that naringenin and luteolin could bind IGF1R, and CETSA and DARTS provided experimental support for IGF1R engagement. Both compounds engaged IGF1R and suppressed IGF-1/IGF1R signaling, thereby reducing PI3K/AKT phosphorylation and the expression of Cyclin D1, BCL2, PCNA, and Ki67. In summary, luteolin and naringenin are potential active constituents of GLTL that suppress proliferation and promote apoptosis in PDGF-BB-stimulated PASMCs in vitro, an effect associated with suppression of IGF1R/PI3K/AKT signaling.

## 1. Introduction

As a progrenssive and fatal disease, pulmonary arterial hypertension (PAH) is characterized by abnormal pulmonary vascular remodeling and persistently elevated pulmonary arterial pressure. The diagnosis of PAH is established when right heart catheterization demonstrates a mean pulmonary artery pressure of more than 20 mmHg in the presence of a pulmonary arterial wedge pressure ≤ 15 mmHg and a pulmonary vascular resistance > 2 Wood units. However, non-specific early symptoms such as dyspnea, fatigue, and chest pain, coupled with complex diagnostic procedures, often lead to delayed diagnosis. Approximately 22,000 deaths worldwide each year are attributed to PAH, which poses a significant public health burden [1,2]. Although existing targeted therapies can improve hemodynamic parameters and alleviate clinical symptoms [3,4], the 5-year survival rate of PAH patients is still only about 57%, largely attributed to sustained disease progression and poor prognosis [5,6]. In addition, due to the limitations of current conventional therapies in reversing vascular remodeling and prolonging survival, the disability and mortality rates of PAH patients remain high [7].

The multi-target collaborative intervention strategy has emerged as a key approach to breaking through therapeutic bottlenecks in PAH, as it simultaneously regulates the core pathological processes of PAH, such as vasoconstriction, anti-proliferation, antioxidant activity, and immune regulation [8,9,10,11]. It is worth noting that herbal medicines and their herbal formulas have unique therapeutic potential in difficult-to-treat diseases including various stubborn lung diseases based on the characteristics of “multi-component, multi-target” effects [12,13]. As part of our continuing study to identify effective agents for PAH from natural herbal medicines, we previously demonstrated that the *Guilou Tongluo* Formula (GLTL, Table S1), an empirically validated herbal formula, exerted favorable therapeutic efficacy in patients with pulmonary hypertension associated with chronic obstructive pulmonary disease [14], supporting its relevance to pulmonary vascular disease. However, the material basis and potential mechanisms by which GLTL exerts therapeutic effects on PAH are still poorly understood and require further research. In addition, bioinformatics analysis and network pharmacology analysis based on multi omics datasets from online public databases are effective methods for comprehensively exploring the potential molecular mechanisms and active substances of herbs [15,16,17]. Therefore, this study used liquid chromatography–mass spectrometry to identify the components in GLTL, and combined them with the blood components in the database for subsequent active ingredient research. Based on comprehensive multi-omics strategies and bioinformatics analysis, drug disease-related mechanism targets were identified. Compounds that can stably bind to the targets were predicted through computational simulations and experimentally validated, thereby systematically elucidating the molecular mechanism and active substances of GLTL against PAH. This will be beneficial for further quality control and modern formulation development.

The existing targeted drugs mainly act on pathways such as endothelin and prostacyclin, essentially correcting vascular imbalance, but it is difficult to reverse the already solidified vascular remodeling. Recent studies have shown that the insulin-like growth factor 1 receptor (IGF1R) and its downstream PI3K/AKT cascade are located at the intersection of cell proliferation, survival, and metabolic reprogramming, and are common downstream bottlenecks for multiple receptor tyrosine kinase signaling, involved in the abnormal proliferation of PASMC and the suppression of apoptosis [18,19]. In animal experiments, smooth muscle specific selective inhibition of PI3K catalytic subunit p110α can not only prevent but also reverse established pulmonary vascular remodeling and pulmonary arterial hypertension [18]; the clinical cohort also showed that circulating levels of IGFBP2, IGFBP4, and IGFBP7 were independently correlated with PAH severity and survival rate [20,21,22]. Therefore, elucidating the exact role of the IGF1R/PI3K/AKT axis in PAH and its intervention value in regulating this pathway is of great significance for breaking through existing treatment bottlenecks.

## 2. Results

### 2.1. GLTL Inhibits PDGF-BB-Induced Aberrant Proliferation and Migration of PASMCs and Promotes Apoptosis

**Figure 1:**
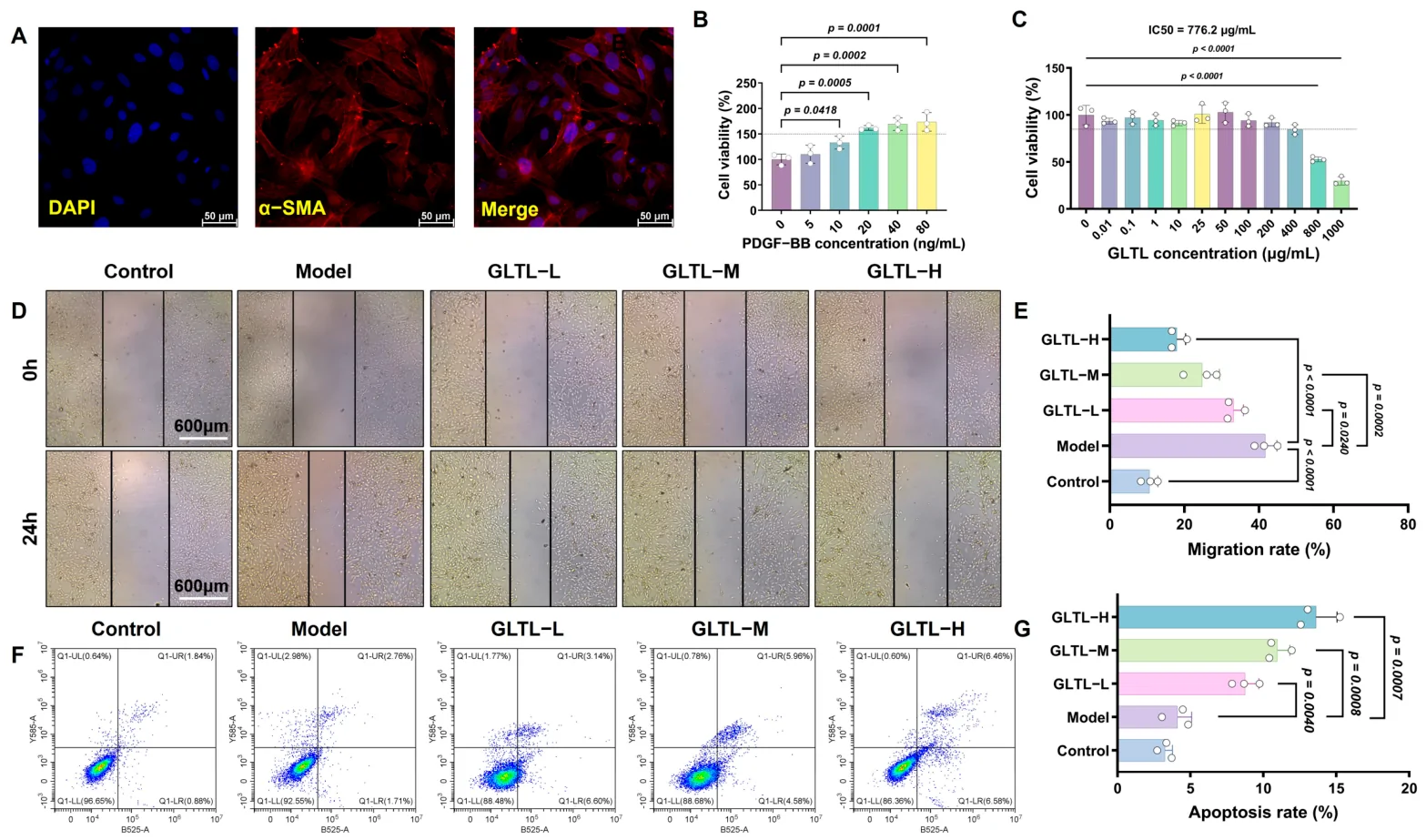
GLTL suppresses PDGF-BB-induced PASMCs proliferation and migration, and promotes apoptosis in PASMCs. (**A**) Immunofluorescence identification of α-SMA in primary PASMCs. (**B**) Determination of the optimal PDGF-BB concentration for inducing PASMCs proliferation. (**C**) Dose–response relationship and IC50 value of GLTL on PASMCs viability. (**D**) Representative im-ages of the scratch assay. (**E**) Quantitative analysis of GLTL inhibition on PDGF BB induced migration in PASMCs. (**F**) Representative flow cytometry plots. (**G**) Apoptosis rate statistics of GLTL induced apoptosis in PASMCs (*n* = 3). Data are presented as mean ± SD. Exact *p* values indicated in the figure. *p* < 0.05 was considered statistically significant. All statistical comparisons were made relative to the Model group.

Primary rat PASMCs were isolated and characterized by α-smooth muscle actin (α-SMA) and calponin immunofluorescence staining to confirm cell identity prior to functional assessment of the GLTL formula (Figure 1A and Figure S1A). Cell Counting Kit-8 (CCK-8) assays revealed a positive correlation between PASMC proliferative activity and PDGF-BB concentration, with the most pronounced increase observed at 20 ng/mL, which was therefore selected for subsequent modeling (Figure 1B). In addition, based on CCK-8 assays, the representative results showed that the half-maximal inhibitory concentration (IC50) value of GLTL extracts for PASMCs was 776.2 μg/mL (Figure 1C). Therefore, based on the IC_50_ value, three concentrations—100 μg/mL (1/8 IC_50_), 200 μg/mL (1/4 IC_50_), and 400 μg/mL (1/2 IC_50_)—were selected as the testing concentrations for GLTL extracts in the following experiments. The anti-migratory effect of GLTL was examined by wound-healing assay. Relative to the control group, the model group exhibited a marked elevation in wound closure percentage, indicating enhanced migratory capacity, whereas GLTL treatment markedly suppressed this effect (Figure 1D,E). Flow cytometric analysis of apoptosis revealed that PDGF-BB stimulation alone did not significantly alter the apoptotic rate, whereas GLTL formula treatment increased apoptosis (Figure 1F,G).

**Figure 2:**
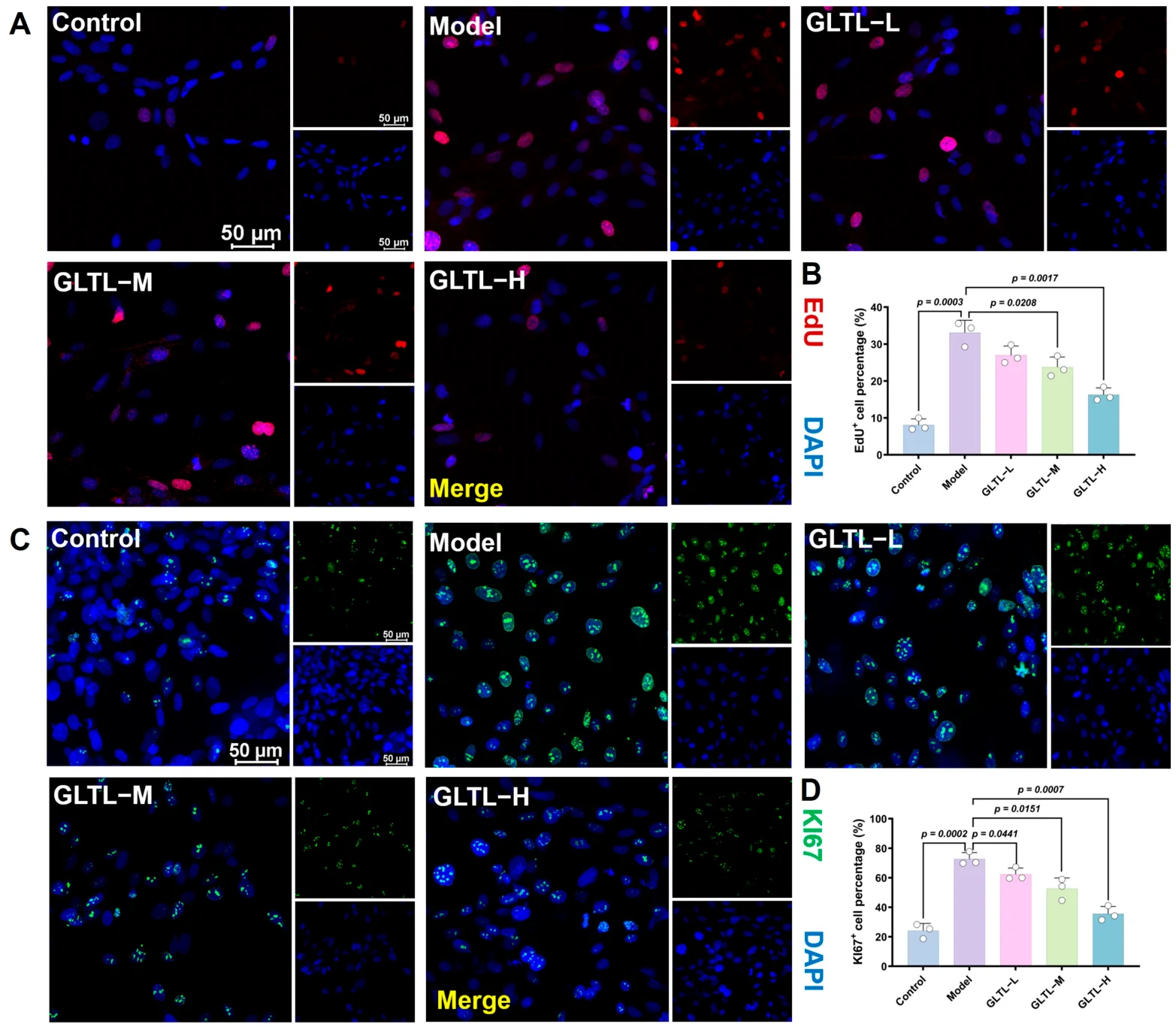
Validation of the inhibitory effect of GLTL on PDGF-BB-induced PASMCs proliferation. (**A**) EdU incorporation assay detecting GLTL-mediated inhibition of PASMCs proliferation. (**B**) Quantitative analysis of EdU incorporation. (**C**) Ki67 immunofluorescence staining evaluating the effect of GLTL on PASMCs proliferation. (**D**) Quantitative analysis of Ki67 positive cells (*n* = 3). Data are presented as mean ± SD. Exact *p* values are indicated in the figure. All statistical comparisons were made relative to the Model group.

EdU incorporation assays further demonstrated that the EdU-positive cell rate was significantly elevated in the model group relative to the normal group, indicative of active proliferation. It is worth noting that the EdU positivity rate in the GLTL treatment group showed a dose-dependent decreasing trend, with the high-dose group showing the most significant decrease (Figure 2A,B). Consistent with this, Ki67 immunofluorescence staining confirmed that the number of Ki67 positive cells in GLTL treatment group was significantly reduced compared to the model group (Figure 2C,D). In summary, the GLTL formula can effectively inhibit the abnormal proliferation and migration of PASMCs induced by PDGF-BB, and promote cell apoptosis, demonstrating its multi-target regulatory ability.

### 2.2. Screening of Disease-Associated Genes

**Figure 3:**
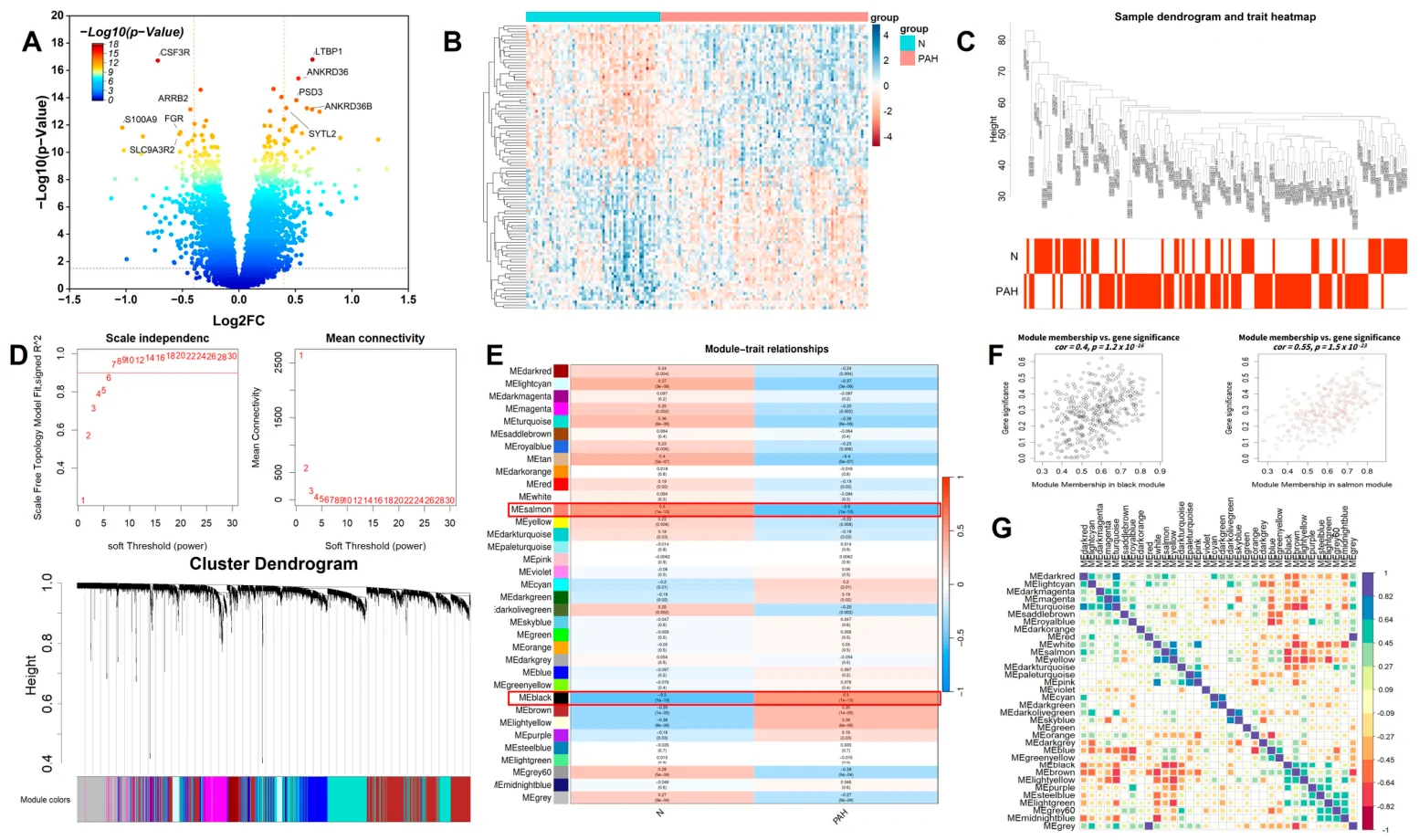
Integrated transcriptomic analysis of PAH combining differential expression analysis and WGCNA. (**A**) Volcano plot of differentially expressed genes (DEGs). (**B**) Heatmap of the DEGs. (**C**) Sample clustering dendrogram and trait heatmap for WGCNA module-trait association analysis. (**D**) Soft-thresholding power evaluation and hierarchical clustering tree construction for network topology optimization. (**E**) Heatmap of module eigengene-trait associations. (**F**) Scatter plot of the correlation between members within the module and gene expression. (**G**) Network heatmap of inter-module correlations revealing functional crosstalk landscapes among co-expression modules. This disease group includes pulmonary hypertension with mixed etiology, labeled as PAH in the figure (see Section 4.3 and Table S4).

After batch effect correction, the expression data of 20,230 genes were retained (Figure S1). Differential expression analysis was performed with a threshold of |log_2_FC| > 0.4, and 232 significantly differentially expressed genes (DEGs) were identified (Figure 3A,B). Weighted Gene Co-expression Network Analysis (WGCNA) was conducted to capture weak but continuously changing genes. The selected soft threshold was 7, and the corresponding scale-free topological fitting index reached 0.9. The co-expression network constructed from this included 35 gene modules (Figure 3C,D). Among them, MEblack (396 genes) and MEsalmon (280 genes) had the strongest association with disease phenotype (*r* = 0.5, *p* = 1 × 10^−10^, Figure 3E). The correlation scatter plot showed robust correlation coefficients and highly significant *p*-values, supporting its biological relevance (Figure 3F). The strong correlation between MEblack and MEsalmon further suggested their potential importance in the pathogenesis of pulmonary vascular remodeling (Figure 3G). Subsequently, DEGs and WGCNA module genes were merged, yielding 859 potential disease-associated genes for subsequent analysis.

### 2.3. Chemical Constituents of GLTL, Prediction of Key Genes and Pathways

**Figure 4:**
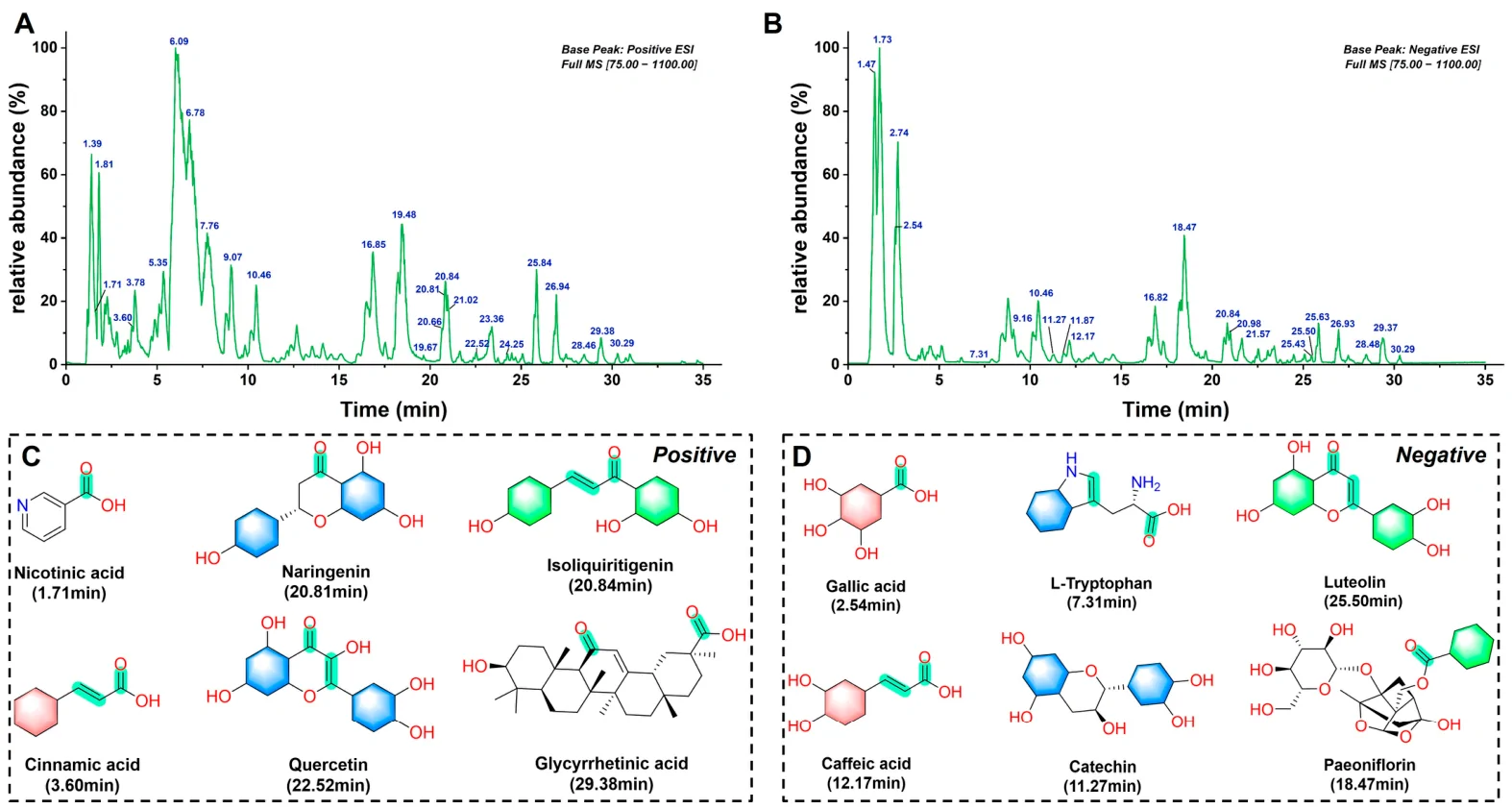
Chemical constituents profiling of the GLTL by UHPLC-Q Exactive MS. (**A**) Base peak chromatograms of the GLTL extract in the positive ion mode. (**B**) Base peak chromatograms of the GLTL extract in the negative ion mode. (**C**) Identification of representative compounds and their retention time in UHPLC-Q Exactive MS positive ion mode. (**D**) Identification of representative compounds and their retention time in UHPLC-Q Exactive MS negative ion mode.

**Figure 5:**
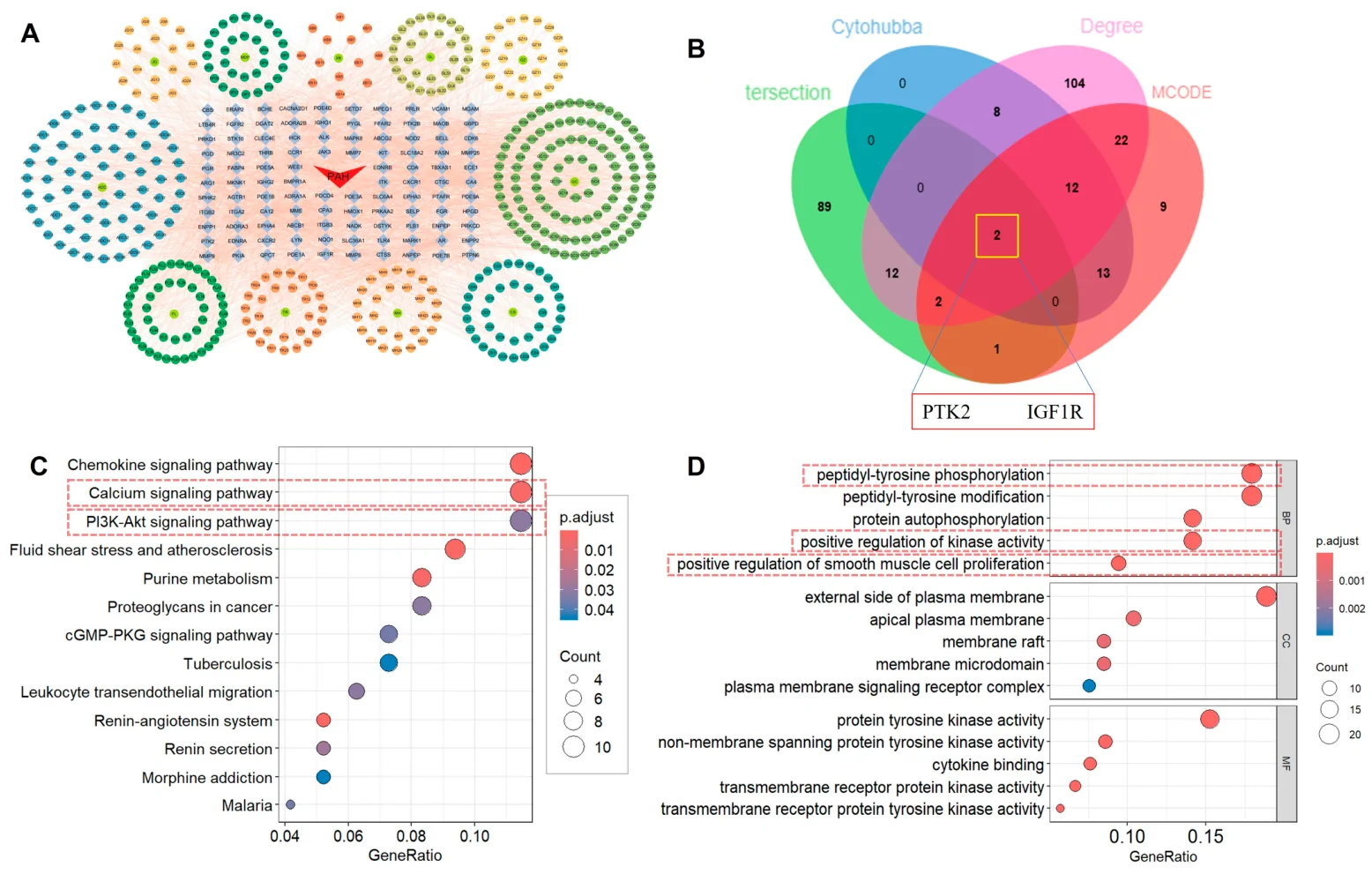
Identification of potential key targets for multi-components of the GLTL intervention in pulmonary hypertension. (**A**) Drug-ingredient-disease-target network. (**B**) Venn diagram of genes identified by multiple methods based on drug-disease gene intersection, CytoHubba, Degree, and MCODE. (**C**) KEGG enrichment bubble plot of the intersecting genes. (**D**) GO enrichment analysis bubble plot of the intersecting genes.

UHPLC-Q Exactive mass spectrometry identified 125 compounds in the GLTL formula (Figure 4, Table 1). Combined with database mining, 438 unique components were obtained (Table S2). Target prediction and database supplementation yielded 1233 drug target genes. Intersecting these with disease-associated genes produced the “Herb-Compound-Disease-Target” network (Figure 5A). After merging disease targets and drug targets, network analysis was performed. From this network, genes were identified using three complementary strategies: degree > 2 × the median, MCODE top-2 modules, and cytoHubba maximal clique centrality (MCC) and maximum neighborhood component (MNC) algorithms (Figure S2A–C). The hub genes identified were IGF1R and protein tyrosine kinase 2 (PTK2), which showed strong correlations with the disease (Figure 5B).

Kyoto Encyclopedia of Genes and Genomes (KEGG) pathway enrichment analysis of the key gene set revealed significant associations with signaling pathways including Chemokine, Calcium, PI3K/AKT, and Renin-Angiotensin system (Figure 5C and Figure S2D,F). According to Gene Ontology (GO) term enrichment, the core genes were mainly linked to biological process terms (protein autophosphorylation, peptidyl tyrosine phosphorylation and modification), cellular component terms (outer plasma membrane leaflet, membrane rafts, and microdomains), and molecular function terms (protein tyrosine kinase activity, non-receptor tyrosine kinase activity, and transmembrane receptor kinase activity) (Figure 5D and Figure S2E,G). These results reflect the characteristics of GLTL formula as a multi-component and multi-target intervention.

### 2.4. Immune Infiltration Analysis Reveals Changes in the Pulmonary Hypertension Microenvironment

Immune infiltration analysis was carried out on the combined expression profile dataset to estimate immune cell proportions in lung specimens, after which samples were categorized accordingly. The results revealed differences in estimated cell proportions between the disease and control (N) groups (Figure S3A). Significantly different cell types included neutrophils, monocytes, plasma cells, resting mast cells, etc., suggesting their potential involvement in the immunopathological process of pulmonary hypertension (Figure S3B). The correlation plot revealed associations among significantly different cell types, including plasma cells and monocytes, M0 macrophages and resting memory CD4+ T cells, resting mast cells, neutrophils, and monocytes (Figure S3C). Pearson correlation analysis was performed to assess the association between intersection gene transcript levels and immune infiltration abundance. The heatmap indicated that neutrophils, monocytes, etc., all correlated strongly with the hub genes, a pattern that mirrored the trends seen across the entire gene set (Figure S3D).

### 2.5. Single-Cell RNA Sequencing (scRNA-seq) Analysis Reveals Cellular Heterogeneity and PI3K/AKT Pathway Activity in PAH Lung Tissue

**Figure 6:**
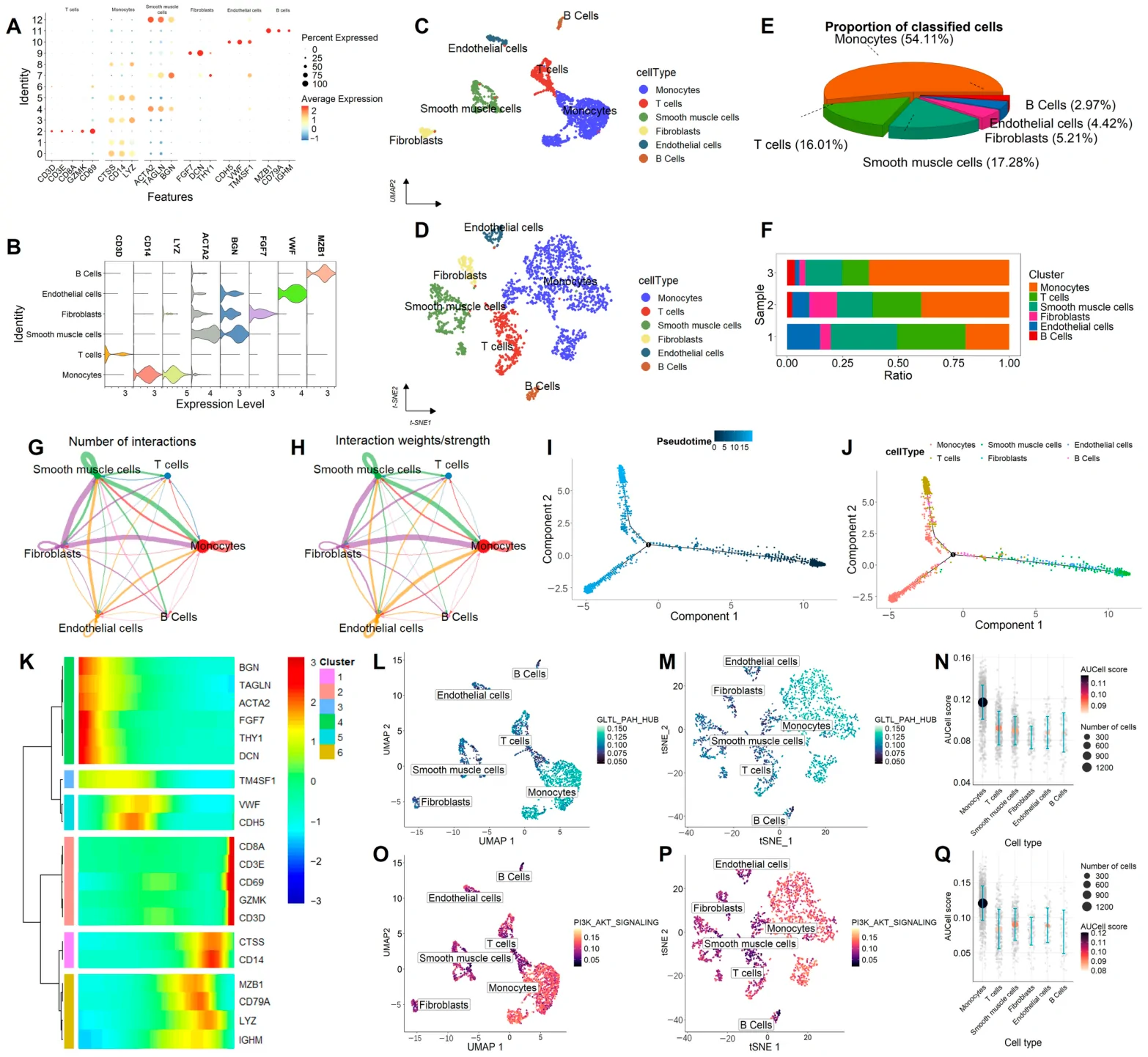
Single-cell transcriptomic analysis reveals cellular heterogeneity, microenvironmental communication, differentiation trajectories, and gene-set activity distribution patterns in lung tissues. (**A**,**B**) Identification and annotation of cell subclusters based on characteristic marker gene expression. (**C**,**D**) Nonlinear dimensionality reduction (uniform manifold approximation and projection (UMAP)/t-distributed stochastic neighbor embedding (t-SNE)) mapping of single-cell data revealing the distributions of six major functional cell subclusters. (**E**,**F**) Proportional composition and abundance changes in different cell subclusters across samples. (**G**,**H**) Inferred intercellular ligand-receptor interaction networks showing communication frequency (left, cell abundance) and interaction strength (right, weighted probability). (**I**,**J**) Pseudotime trajectory analysis of cell lineage differentiation and inference of fate decisions at key branching nodes. (**K**) Heatmap of marker gene expression modules dynamically regulated along the pseudotime trajectory. (**L**–**N**) Single-cell level AUCell score mapping of core gene module activities. (**O**–**Q**) Single-cell level AUCell score mapping of PI3K-AKT signaling axis activity, UMAP (left) and tSNE (right) dual-projection comparative validation.

After quality control, the top 2000 highly variable genes (HVGs) were selected for principal component analysis (PCA), and the top 10 principal components (PCs) were selected for downstream analysis (Figure S4A–E). Using the k-nearest neighbor algorithm based on Euclidean distance (resolution of 0.8) for clustering, cells were divided into 12 subgroups (Figure S4F–I). By manually annotating classic marker genes, six cell subtypes were identified: monocytes, T cells, smooth muscle cells (SMCs), fibroblasts, endothelial cells (ECs), and B cells. Among them, monocytes were the most abundant population, followed by smooth muscle cells and T cells (Figure 6A–F and Figure S4J). Cell–cell communication analysis showed significant interactions between smooth muscle cells, monocytes, and fibroblasts, suggesting that smooth muscle cells may be the core nodes in the intercellular communication network (Figure 6G,H and Figure S5A,B). Further analysis of the pseudotime trajectory within this PAH cohort indicated that SMCs, fibroblasts, and ECs predominated at the early stages of the trajectory, whereas immune cells became progressively more abundant at later pseudotime (Figure 6I–K and Figure S5C,D); due to the lack of healthy control donors in this study, these trajectories and enrichment results should be considered descriptive findings rather than direct evidence of disease-specific activation. Importantly, both AUCell and AddModuleScore analyses showed that the core gene module and the PI3K/AKT signaling pathway were enriched in SMCs, second only to monocytes, identifying these two cell types as candidate cellular sources of the proliferative signal within PAH tissue (Figure 6L–Q and Figure S5E–G).

### 2.6. Identification of IGF1R as a Robust Candidate Target and Evaluation of Its Association with Disease at the Tissue Level

**Figure 7:**
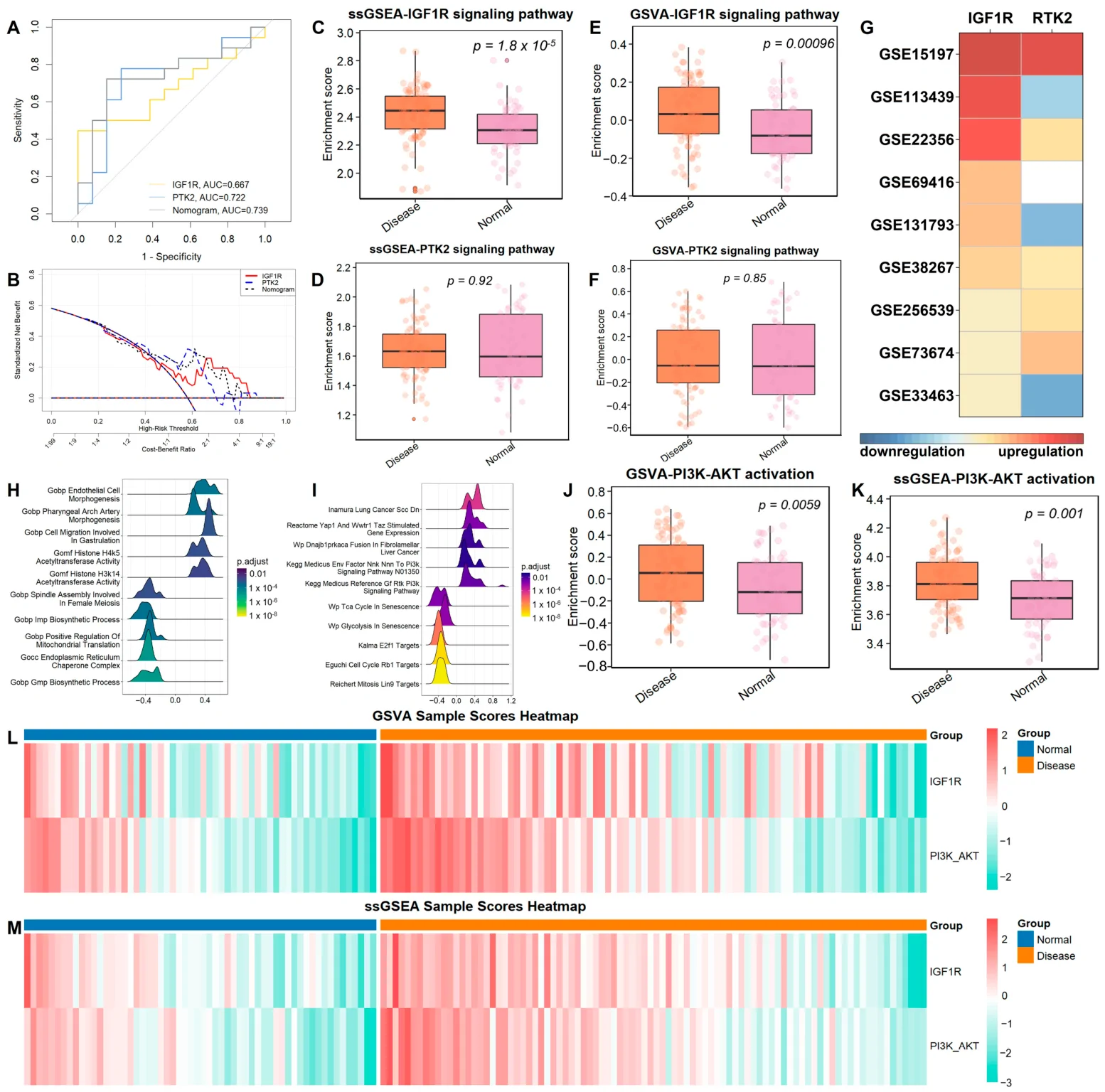
Association of candidate targets PTK2 and IGF1R with disease status and validation of their signaling pathway activities. (**A**,**B**) ROC curve and decision curve analysis of the multivariable tissue-level association model incorporating PTK2 and IGF1R. (**C**,**D**) Group comparisons of ssGSEA enrichment scores for IGF1R and PTK2 signaling pathways. (**E**,**F**) Group comparisons of GSVA enrichment scores for IGF1R and PTK2 signaling pathways. (**G**) Cross-platform validation of IGF1R and PTK2 expression across multiple independent datasets. (**H**,**I**) Ridge plots of single-gene GSEA for IGF1R: GO terms (left) and KEGG pathways (right). (**J**,**K**) Group comparison of GSVA and ssGSEA enrichment scores for the PI3K/AKT signaling pathway. (**L**,**M**) GSVA and ssGSEA heatmaps of sample-level co-activation patterns between IGF1R and PI3K-AKT pathways. Data are presented as mean ± SD. Exact *p* values indicated in the figure.

In this study, a multivariable logistic regression model was constructed and a nomogram was developed to model the association between candidate target-gene expression and disease status in lung tissue (Figure S6A). The ROC curve demonstrated moderate discriminative ability (area under the curve, AUC = 0.739, Figure 7A). In addition, decision curve analysis (DCA) showed that the multivariable model that included PTK2 and IGF1R demonstrated favorable net benefit across a wide spectrum of threshold probabilities in this tissue-level model (Figure 7B), indicating that PTK2 and IGF1R are mechanistically associated with pulmonary hypertension.

Gene set variation analysis (GSVA) and single-sample gene set enrichment analysis (ssGSEA) were further employed to evaluate pathway-level enrichment of IGF1R and PTK2. IGF1R was significantly enriched in disease samples compared with normal controls (Figure 7C,E). Notably, PTK2 enrichment scores did not reach statistical significance between groups, and the distribution patterns showed no significant difference (Figure 7D,F). This finding was further validated by examining the expression trends of the two genes across multiple datasets, in which IGF1R exhibited a more stable pattern, demonstrating greater cross-dataset robustness as a tissue-level disease-associated marker (Figure 7G).

Therefore, single-gene Gene Set Enrichment Analysis (GSEA) was performed on IGF1R to explore its biological functions via GO and KEGG enrichment analyses. Single-gene GSEA revealed that IGF1R high-expression-associated genes were predominantly enriched in pathways related to cell cycle regulation, metabolic reprogramming, protein homeostasis, epigenetic remodeling, and developmental morphogenesis, with significant enrichment also observed in the PI3K/AKT signaling pathway (Figure 7H,I and Figure S6B,C). Subsequent GSVA and ssGSEA analyses consistently showed that PI3K/AKT pathway enrichment scores were significantly elevated in the disease group compared with the normal group (Figure 7J,K), and the same trend was observed in the validation dataset (Figure S6D–G), indicating aberrant activation of this pathway in pulmonary hypertension. Heatmaps further illustrated the co-activation patterns of these two pathways at the individual sample level (Figure 7L,M and Figure S6H–I).

### 2.7. GLTL Inhibits PASMCs Proliferation and Promotes Apoptosis via IGF1R/PI3K/AKT Pathway

**Figure 8:**
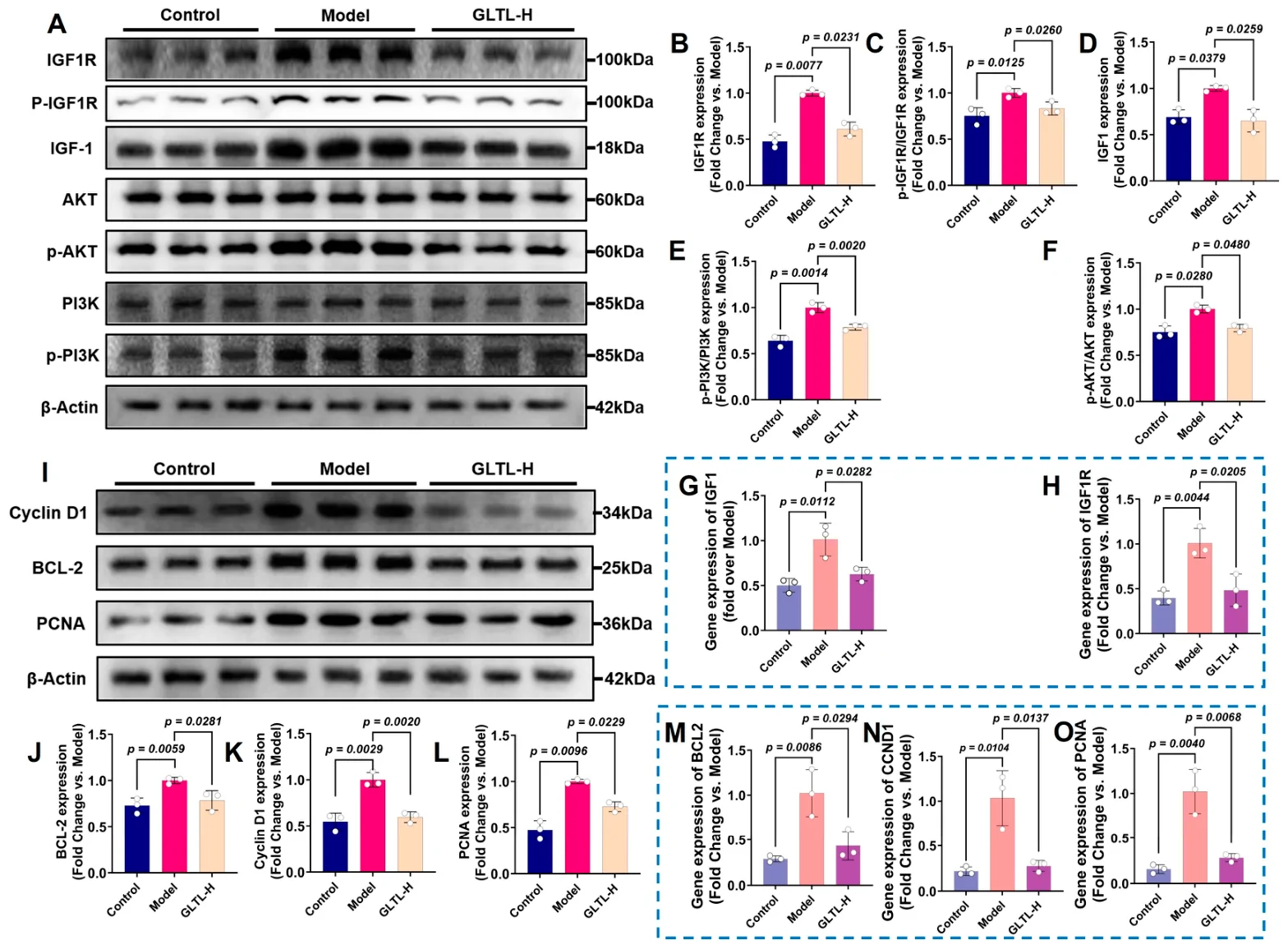
GLTL modulates IGF1R and PI3K-AKT activities. (**A**–**F**) Western blot analysis of the effects of GLTL on the expression of core proteins of the IGF1R and PI3K/AKT pathways in PASMCs. (**G**,**H**) qPCR analysis of GLTL mediated regulation of IGF1R and IGF1 mRNA expression levels. (**I**–**L**) Western blot analysis of the effects of GLTL on the expression of proliferation and anti-apoptosis related proteins in PASMCs. (**M**–**O**) qPCR analysis of GLTL mediated regulation of BCL2, CCND1, and PCNA mRNA expression levels (*n* = 3). Data are presented as mean ± SD. Exact *p* values indicated in the figure. All statistical comparisons were made relative to the Model group.

Based on bioinformatic findings implicating the IGF1R/PI3K/AKT axis in pulmonary vascular remodeling, we next interrogated this pathway in PASMCs. Immunoblotting demonstrated that, versus the control group, the model group showed substantially higher protein abundance of IGF1R and its ligand IGF-1, accompanied by markedly increased phosphorylation levels of PI3K and AKT. Notably, high-dose GLTL significantly attenuated these PDGF-BB-induced alterations in vitro, suppressing IGF1R and IGF-1 expression and reducing IGF1R/PI3K/AKT phosphorylation (Figure 8A–F). qPCR analysis further confirmed that GLTL significantly inhibited IGF1R and IGF-1 at the mRNA level (Figure 8G,H). Having confirmed pathway activation in the model, we next examined the downstream effectors including Cyclin D1, BCL2, and PCNA. The results showed that both protein and transcript levels of Cyclin D1, BCL2, and PCNA were notably increased in the model group (Figure 8I–O). In summary, these findings demonstrated that the GLTL formula exerts its anti-proliferative and pro-apoptotic effects, at least in part, through suppression of the IGF1R/PI3K/AKT signaling pathway.

### 2.8. Computational and Experimental Validation of IGF1R-Targeting Constituents from GLTL via Molecular Docking, Dynamics, and CETSA/DARTS Assays

**Figure 9:**
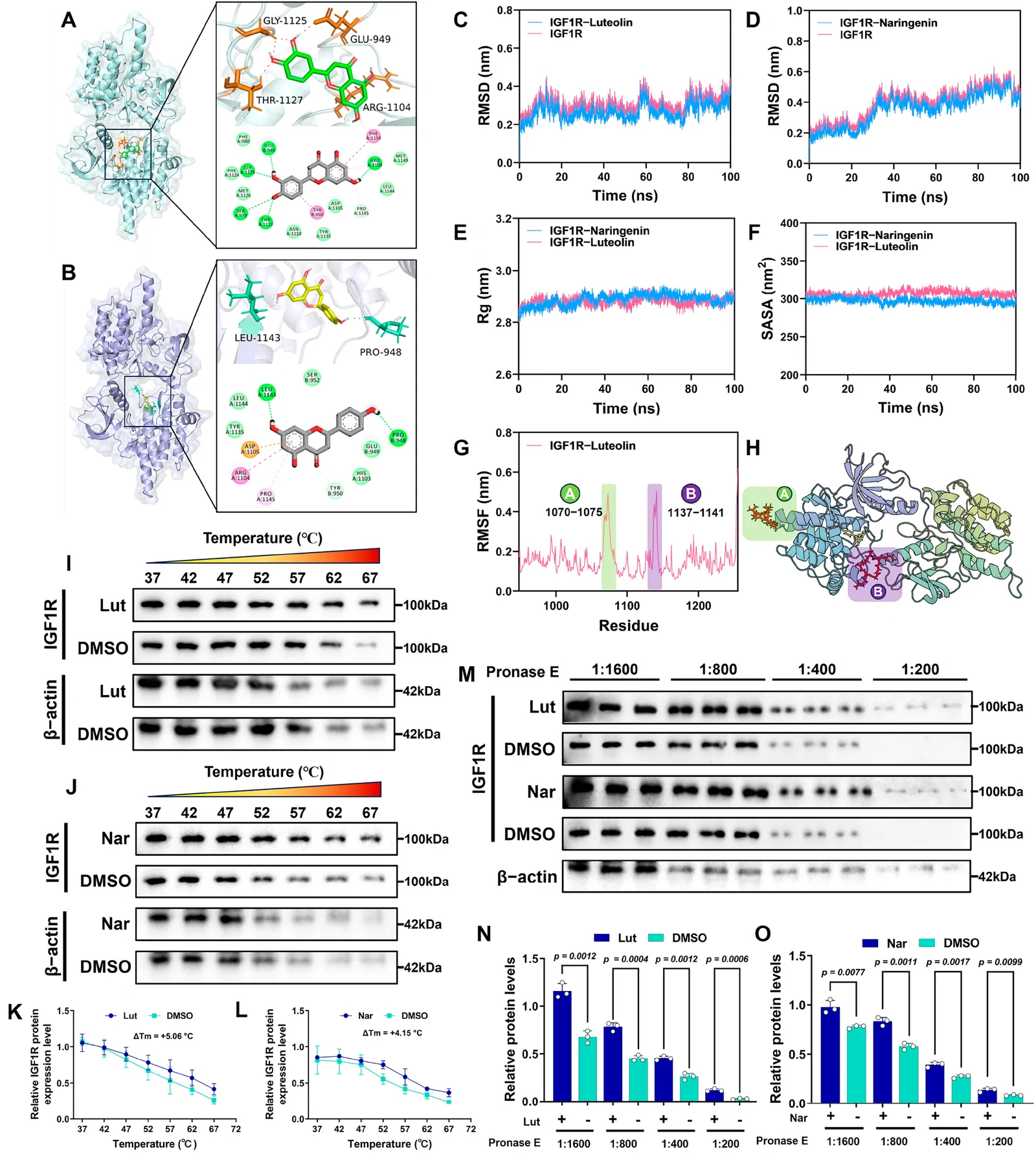
Integrated in silico prediction and DARTS/CETSAs support the engagement of IGF1R by bioactive ingredients of the GLTL. (**A**,**B**) Molecular docking binding modes and key intermolecular interactions of luteolin (Lut, (**A**)) and naringenin (Nar, (**B**)) with IGF1R. (**C**,**D**) Time-dependent RMSD fluctuations of the protein backbone in the luteolin and naringenin complexes tracked by molecular dynamics. (**E**) Evolution of the radius of gyration (Rg) reflecting global conformational compactness of complexes over simulation time. (**F**) Dynamic trajectories of solvent-accessible surface area (SASA) during the simulations. (**G**,**H**) RMSF analysis revealing residue-level flexibility variations within the luteolin complex; A and B represent residue ranges. (**I**–**L**) CETSA detecting thermal stability shifts of IGF1R upon binding with luteolin and naringenin. (**M**–**O**) DARTS assay showing the effect of luteolin and naringenin on Pronase E-mediated degradation of IGF1R; band intensities were normalized to the β-actin band at the lowest protease concentration (1:1600). (*n* = 3). Data are presented as mean ± SD. Exact *p* values indicated in the figure.

To identify bioactive components within the GLTL formula capable of targeting IGF1R, molecular docking screening was performed between IGF1R and 10 major compounds derived from the GLTL, based on prior network pharmacology predictions. Docking results revealed that luteolin and naringenin displayed the lowest binding energies and the most favorable binding conformations with IGF1R, suggesting that these two compounds are the most promising IGF1R-targeting candidates within the GLTL formula (Figure 9A,B and Figure S7A–H; Table S3). The contents of the two compounds in the extract were determined by external-standard HPLC (Figure S8A–E). At the GLTL concentrations applied to the cells, the culture medium contained 1.442 and 1.617 μg/mL of luteolin and naringenin, respectively, at 400 μg/mL GLTL, with proportionally lower levels at 200 μg/mL (0.721 and 0.809 μg/mL) and 100 μg/mL (0.361 and 0.404 μg/mL). These amounts are well below the concentrations at which the isolated compounds were tested (Section 2.9).

To further validate the binding stability of luteolin and naringenin with IGF1R, molecular dynamics (MD) simulations were performed. Root-mean-square deviation (RMSD) analysis of the protein backbone demonstrated that both complexes stabilized during the later stages of the simulations, indicating that the systems had reached equilibrium (Figure 9C,D). Further investigation of the radius of gyration and solvent accessible surface area (SASA) indicated that both complexes maintained favorable overall conformational compactness and structural integrity throughout the trajectories (Figure 9E,F). Free energy landscape analysis revealed that both complexes adopted well-defined low-energy conformations, indicating that the systems are thermodynamically stable (Figure S7I,J). Additionally, the two complexes preserved at least two hydrogen bonds over the entire simulation period (Figure S7K,L). Root-mean-square fluctuation (RMSF) analysis showed that ligand binding reduced the flexibility of residues across most regions, with no connectivity observed between the highly flexible regions and the ligand molecules, further corroborating that luteolin and naringenin stabilize IGF1R conformation upon binding (Figure 9G,H and Figure S7M,N).

To test these interactions in a cellular context, CETSA and DARTS were performed. CETSA showed that both compounds stabilized IGF1R against heat-induced denaturation (Figure 9I–L): the melting temperature (Tm) of IGF1R was 57.95 ± 2.36 °C in luteolin-treated and 57.35 ± 2.85 °C in naringenin-treated cells, compared with 52.89 ± 4.52 °C and 53.20 ± 4.25 °C in the corresponding DMSO controls, giving ΔTm values of +5.06 °C and +4.15 °C, respectively. In the DARTS assay, luteolin and naringenin attenuated the loss of IGF1R signal at each Pronase E concentration relative to vehicle-treated samples (Figure 9M–O), whereas the non-target protein β-actin was progressively degraded with increasing protease concentration in all groups, suggesting that this effect did not reflect a general inhibition of proteolysis. Because no protease-free control lane was included, band intensities were normalized to β-actin at the lowest protease concentration (1:1600), where proteolysis is minimal, and the DARTS results are therefore interpreted as a relative measure of protease protection rather than as a stand-alone demonstration of binding. Taken together, CETSA and DARTS are consistent with intracellular engagement of IGF1R by luteolin and naringenin, providing a basis for investigating their pharmacological effects.

### 2.9. Luteolin and Naringenin Individually Inhibit PDGF-BB-Induced PASMC Proliferation and Migration and Promote Apoptosis

**Figure 10:**
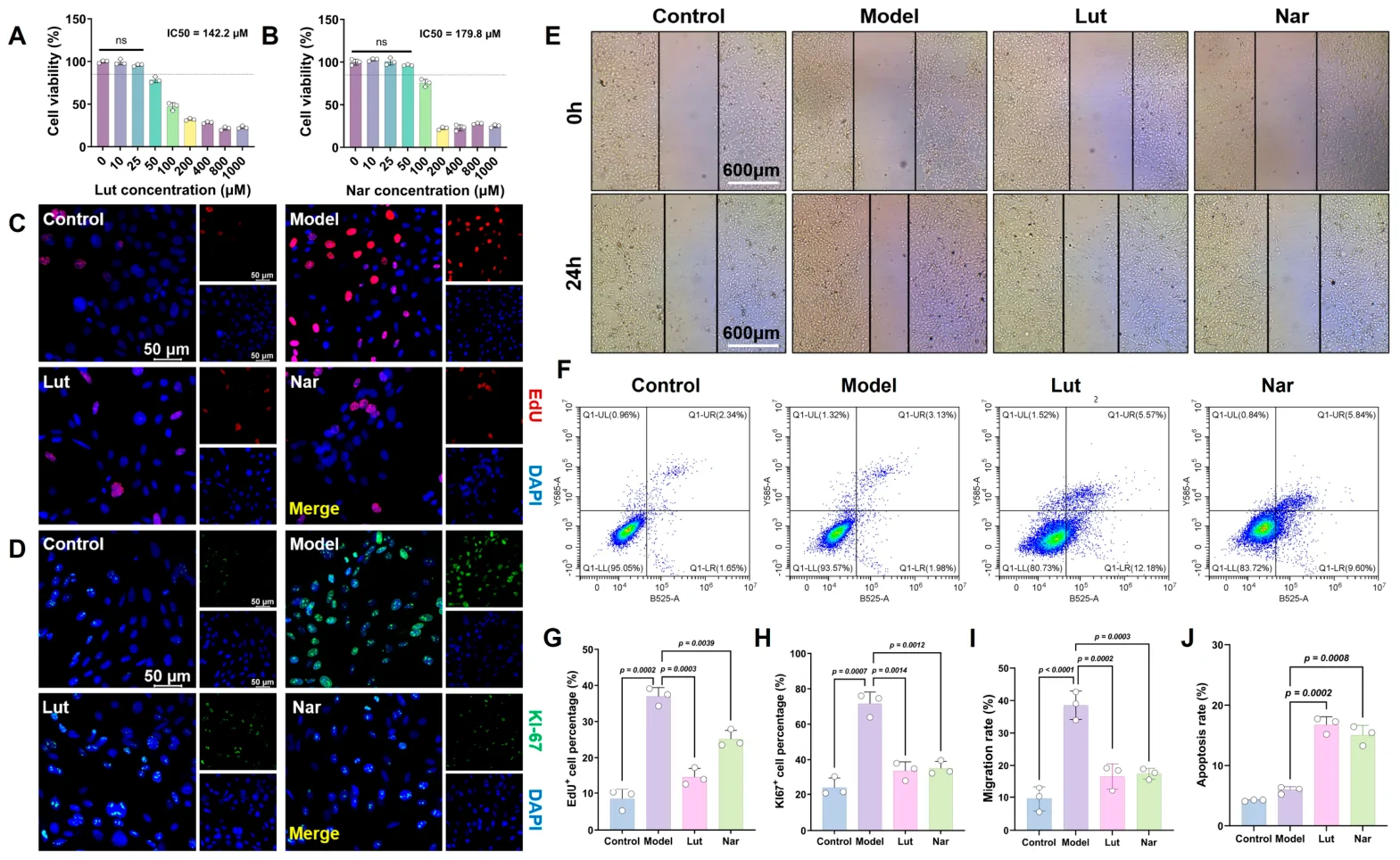
Effects of luteolin and naringenin on PDGF-BB-induced proliferation, migration, and apoptosis of PASMCs. (**A**,**B**) Activity evaluation and IC50 value of luteolin (Lut, (**A**)) and naringenin (Nar, (**B**)) on PASMCs. (**C**) EdU incorporation assay detecting the inhibitory effects of the two compounds on PASMCs proliferation. (**D**) Ki67 immunofluorescence staining evaluating the effects of the two compounds on PASMCs proliferation. (**E**) Wound-healing assay detecting the inhibitory effects of the two compounds on PDGF-BB-induced PASMCs migration. (**F**) Flow cytometry detecting the effects of the two compounds on PDGF-BB-induced PASMCs apoptosis. (**G**,**H**) Quantitative analysis of EdU (**G**) and Ki67 (**H**) immunofluorescence positive rates. (**I**) Quantitative analysis of PASMCs migration rate following treatment with the two compounds. (**J**) Quantitative analysis of PASMCs apoptotic rate following treatment with the two compounds (*n* = 3). Data are presented as mean ± SD. Exact *p* values indicated in the figure. All statistical comparisons were made relative to the Model group.

Having established that the GLTL formula effectively regulates PASMC function and that its two principal constituents were predicted to bind IGF1R stably, we next evaluated the pharmacological effects of these two main bioactive components individually. Similar to the GLTL extract, the effects of luteolin and naringenin on PASMC cell viability were first evaluated by CCK-8 assay to determine IC50 values (Figure 10A,B). Based on the IC50 values, half of the respective IC50 values (70 μM for luteolin and 90 μM for naringenin) were selected as the testing concentrations for subsequent experiments. At these concentrations the CCK-8 signal of unstimulated PASMCs was reduced by approximately 30% (Figure 10A,B); because CCK-8 reports overall metabolic activity, this reduction cannot be attributed exclusively to pathway-specific inhibition, and a non-specific component cannot be excluded.

EdU assay results revealed that, compared with the model group, both luteolin and naringenin treatment significantly reduced the EdU-positive cell rates (Figure 10C,G). Ki67 immunofluorescence staining further confirmed that both compounds significantly decreased the number of Ki67-positive cells (Figure 10D,H). Wound-healing assays demonstrated that PASMC wound closure rates were markedly reduced following luteolin or naringenin treatment compared with the model group (Figure 10E,I). Flow cytometric analysis revealed that apoptosis rates were significantly elevated in both luteolin- and naringenin-treated groups relative to the model group (Figure 10F,J). In summary, these findings demonstrate that luteolin and naringenin each exert inhibitory effects on PASMC proliferation and migration while promoting apoptosis in vitro, identifying them as representative active constituents that may contribute to the anti-vascular re-modeling activity of the GLTL formula.

### 2.10. Luteolin and Naringenin Inhibit Proliferation and Promote Apoptosis via Suppression of the IGF1/IGF1R Signaling

**Figure 11:**
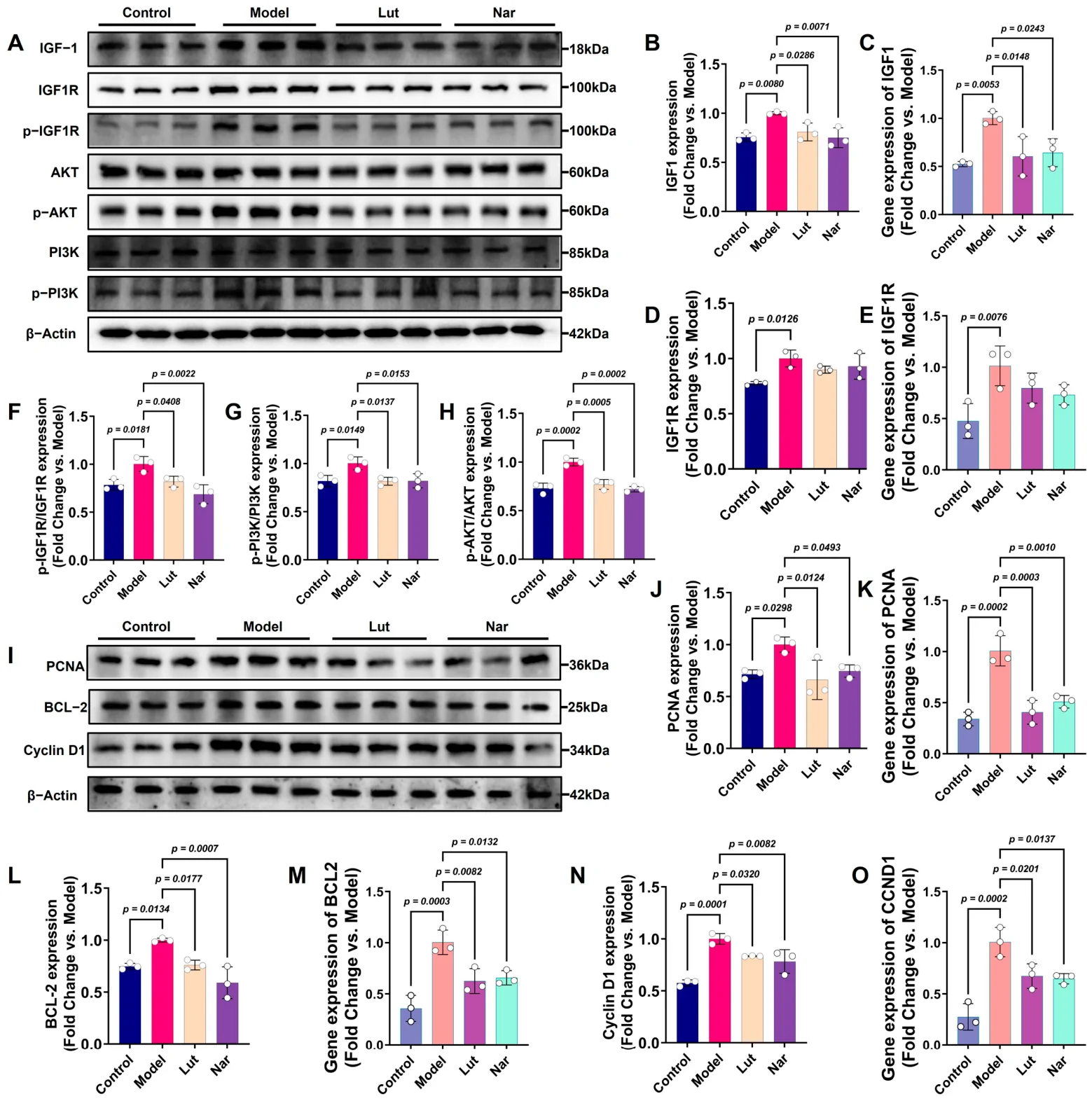
Luteolin and naringenin inhibit PASMCs proliferation via IGF1R/PI3K/AKT signaling axis. (**A**) Western blot analysis of the effects of luteolin and naringenin on IGF1R protein levels and core components of the PI3K/AKT pathway. (**B**–**E**) Quantitative analysis of the effects of the two compounds on the protein expression and gene transcription levels of IGF1 and IGF1R. (**F**–**H**) Quantitative analysis of the effects of the two compounds on the phosphorylation levels of IGF1R and PI3K-AKT proteins. (**I**) Western blot analysis of the effects of luteolin and naringenin on the protein levels of PCNA, BCL2, and cyclin D1. (**J**–**O**) Quantitative analysis of the effects of the two compounds on the protein and mRNA expression levels of proliferation-, cell cycle-, and anti-apoptosis-related markers (*n* = 3). Data are presented as mean ± SD. Exact *p* values indicated in the figure. All statistical comparisons were made relative to the Model group.

Having established that luteolin and naringenin may engage IGF1R and exert therapeutic effects, we further investigated the regulatory effects of these two compounds on downstream signaling of this pathway. Immunoblotting indicated that, relative to the control group, the model group showed a marked increase in protein levels of IGF1R and its ligand IGF1, accompanied by markedly increased phosphorylation levels of PI3K and AKT. Following luteolin or naringenin treatment, IGF1 expression was significantly suppressed, whereas the expression of IGF1R displayed a decreasing tendency that did not reach statistical significance, consistent with their mRNA expression patterns (Figure 11A–E). However, quantitative results demonstrated that both compounds significantly reduced the p-IGF1R/IGF1R, p-PI3K/PI3K, and p-AKT/AKT ratios (Figure 11F–H), indicating marked inhibition of the phosphorylation levels of these proteins. Based on these findings, we further examined the expression of the downstream effector molecules, including Cyclin D1, BCL2, and PCNA. Immunoblotting and qPCR data indicated that the model group had higher protein and mRNA levels of Cyclin D1, BCL2, and PCNA. Both compounds significantly decreased these levels. (Figure 11I–O).

In summary, these findings demonstrate that although luteolin and naringenin have limited effects on total IGF1R protein expression, they significantly suppress IGF1 expression, effectively reduce IGF1R phosphorylation, and block PI3K and AKT phosphorylation and activation, thereby downregulating the proliferation-associated proteins Cyclin D1 and PCNA as well as the anti-apoptotic protein BCL2. These results suggest that both compounds exert their anti-proliferative and pro-apoptotic effects at least in part through inhibition of IGF1R kinase activity and suppressing the IGF-1/IGF1R signaling.

### 2.11. Pharmacological Inhibition of IGF1R/IR Reproduces the Modulatory Effects of Luteolin and Naringenin on PDGF-BB-Induced Proliferation and Apoptosis in PASMCs

**Figure 12:**
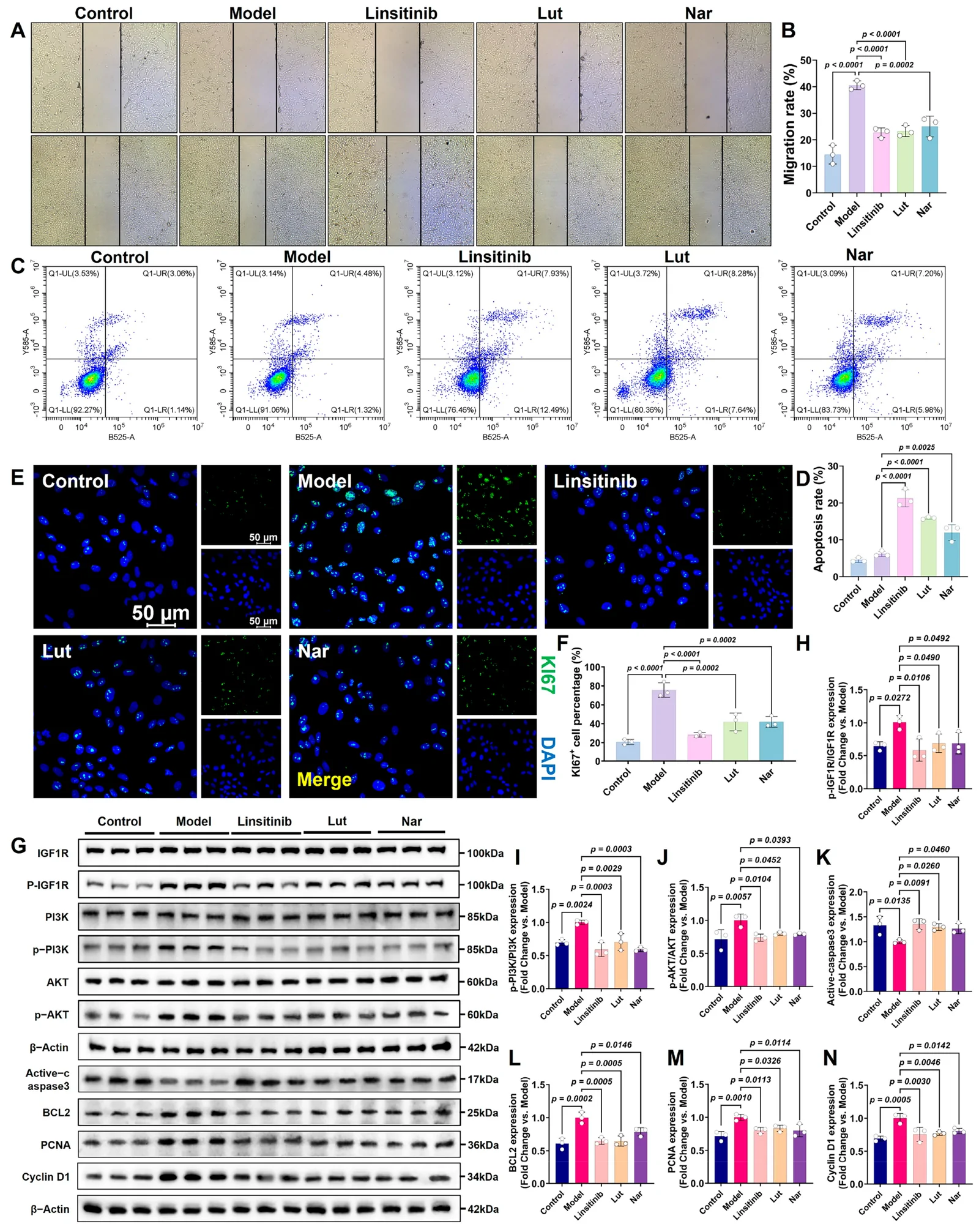
Pharmacological inhibition of IGF1R/IR replicates the effects of luteolin and naringenin in PASMCs. (**A**) Comparison of scratch healing in PDGF-BB-stimulated PASMCs treated with linsitinib. (**B**) Statistical analysis of compound migration rates. (**C**) Flow cytometry analysis of the effect of linsitinib on apoptosis in PDGF-BB-stimulated PASMCs. (**D**) Statistics on apoptosis rates. (**E**,**F**) Ki67 immunofluorescence staining and quantitative analysis following linsitinib treatment. (**G**) Western blot analysis of the effects of the inhibitor and the two compounds on the IGF1R/PI3K/AKT pathway. (**H**–**J**) Quantitative analysis of the p-IGF1R/IGF1R, p-PI3K/PI3K, and p-AKT/AKT ratios. (**K**–**N**) Quantitative Western blot analysis of active-caspase-3, BCL2, PCNA, and cyclin D1 (*n* = 3). Data are expressed as mean ± standard deviation. Exact *p*-values are indicated in the figure. All statistical comparisons were made relative to the Model group.

To further investigate whether the cellular effects of luteolin and naringenin are mediated by IGF1R, we employed a pharmacological loss-of-function approach using linsitinib (OSI-906), a tyrosine kinase inhibitor that blocks both IGF1R and the insulin receptor (IR). First, a CCK-8 assay was performed to determine the concentration-effect relationship of linsitinib in PDGF-BB-stimulated PASMCs, yielding an IC_50_ value of 7.02 μM (Figure S8F). Based on these results, we selected a concentration of 3.51 μM—half the IC_50_ value—for a parallel comparison with the two compounds. In PDGF-BB-stimulated PASMCs, linsitinib inhibited migration and Ki67 expression to an extent comparable to that of luteolin and naringenin and similarly increased the apoptosis rate (Figure 12A–F). At the signaling level, linsitinib significantly reduced the p-IGF1R/IGF1R, p-PI3K/PI3K, and p-AKT/AKT (Figure 12G–J), while simultaneously downregulating the downstream effector molecules PCNA, Cyclin D1, and BCL2, and increasing the levels of active caspase-3 (Figure 12K–N), with effects comparable to those of the two aforementioned flavonoids. Pharmacological blockade of IGF1R/IR kinase activity produced antiproliferative and proapoptotic effects similar to those of luteolin and naringenin, from the cellular phenotype down to receptor phosphorylation and downstream effectors. Because linsitinib inhibits both IGF1R and the insulin receptor, these data indicate that the effects of the two compounds converge on the IGF1R/IR-PI3K/AKT axis, but they do not establish that the compounds act through IGF1R specifically.

## 3. Discussion

In this study, network pharmacology and bioinformatics approaches were integrated to construct an “herbal medicine formula-bioactive components-targets-disease” interaction network based on public pulmonary hypertension transcriptomic datasets and herbal medicines component-related target screening, with the aim of systematically identifying candidate targets of GLTL against PAH. Subsequently, PPI network topology analysis was performed to screen hub genes, which were further characterized by GO/KEGG pathway enrichment analysis, immune infiltration analysis, and scRNA-seq to decipher their expression patterns and regulatory networks in pulmonary vascular resident cells and immune cell subsets. Furthermore, multivariable logistic regression models and nomograms were used to explore the association between hub-gene expression and disease status in lung tissue, and molecular docking combined with MD simulations was employed to assess the binding affinity and stability between representative targets and active components. On the basis of the above computational analyses, systematic in vitro validation of key targets and pathways was conducted through CCK-8 cell viability assays, EdU incorporation assays, Ki67 immunofluorescence staining, wound-healing assays, and flow cytometric detection of apoptosis, coupled with Western blot and qPCR analyses to evaluate the activation status of the core signaling pathways and their downstream effectors at both protein and mRNA levels. This integrated research strategy not only systematically elucidates the main active ingredients and mechanisms of action of GLTL, but also provides a universal methodological paradigm for the systematic pharmacology research of herbal medicine prescriptions. It should be emphasized that the transcriptomic datasets analyzed here represent heterogeneous pulmonary hypertension aetiologies; the bioinformatic signatures are therefore interpreted as convergent vascular-remodeling features shared across these groups, whereas the mechanistic conclusions derived from the PASMC experiments pertain specifically to the PAH context.

**Figure 13:**
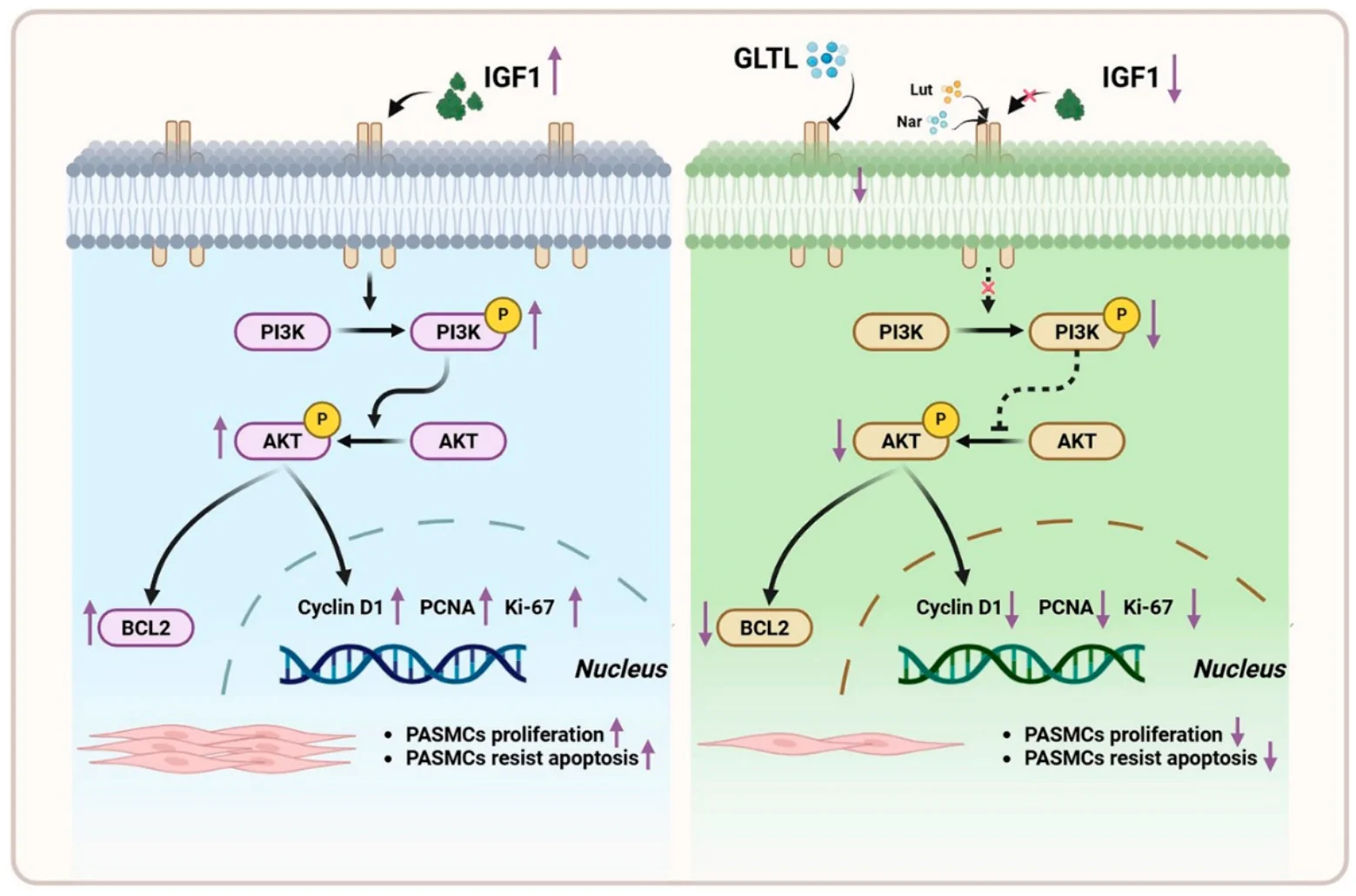
GLTL inhibits abnormal proliferation and promotes apoptosis in PASMCs by inhibiting the IGF1R/PI3K/AKT pathway. Created in BioRender. Dwad, D. (2026) https://BioRender.com/zd12zzu (accessed on 30 September 2026).

The PI3K/AKT pathway was significantly enriched in KEGG analysis, consistent with the GO functional enrichment results, and was therefore selected as a key research focus. Subsequent scRNA-seq analysis revealed that the AUCell and AddModuleScore of this pathway were highly correlated with monocytes and smooth muscle cells, suggesting that this pathway may play critical roles in regulating the functions of these two cell types. IGF1R, a member of the tyrosine kinase family [23], serves as an important upstream regulator of the PI3K/AKT pathway. Through integrated network pharmacology and bioinformatics analyses, we identified IGF1R as a potential core target from PPI network topology screening and pathway enrichment results; its aberrant expression in pulmonary hypertension and close association with the PI3K/AKT pathway further suggested that IGF1R may act as a key regulatory node of this pathway. According to the canonical signaling cascade, IGF1R activates AKT via IRS/PI3K/PIP3/PDK1, which subsequently regulates downstream molecules including GSK3/mTORC1/GLUT, CREB, Caspase-9, and P21/P27, thereby promoting cell proliferation, survival, and metabolism [24]. Our Western blot and qPCR results further confirmed that in the PDGF-BB-induced PASMC model, the phosphorylation levels of IGF1R and downstream PI3K/AKT were significantly elevated, whereas intervention with GLTL and its active components effectively suppressed this activation, downregulated the expression of the proliferation-associated proteins PCNA and Cyclin D1 and the anti-apoptotic protein BCL-2, and concurrently reduced the proportion of Ki67-positive cells (Figure 13). These findings support that aberrant activation of the IGF1R/PI3K/AKT signaling pathway is a key molecular event driving the pathological proliferation, anti-apoptosis, and migration of PASMCs. Moreover, prior reports indicate that PI3K/AKT activation contributes to modulating multiple inflammatory signaling cascades, such as NF-κB [25]. Besides the PI3K/AKT axis, our KEGG enrichment results also revealed significant enrichment of inflammatory and immune-related pathways, such as the Chemokine signaling pathway and the Renin-angiotensin system, which may be associated with the aberrant monocyte enrichment observed in our immune infiltration analysis [26,27]. Collectively, these findings suggest that GLTL may exert its effects through the simultaneous regulation of PI3K/AKT signaling-mediated vascular remodeling and immune-related inflammatory pathways.

This study, through immune infiltration analysis and single-cell data analysis, revealed aberrant enrichment of monocytes in the pulmonary vascular tissues of PAH patients. These infiltrating monocytes may rapidly differentiate into macrophages under the regulation of the local microenvironment, becoming the predominant inflammatory cell type in pulmonary vascular pathology [28,29]. Stimulated monocytes and macrophages secrete high levels of inflammatory cytokines and chemokines, generating a self-reinforcing inflammatory circuit that recruits additional inflammatory cells to the lesion site. This persistent inflammatory microenvironment constitutes a core pathophysiological feature of pulmonary hypertension, which may directly damage vascular cells and promote vascular remodeling [30,31]. Given the central role of monocytes in PAH, intervention strategies targeting their recruitment, activation, or function have become important research directions. Treatment methods may include targeting the CCL2/CCR2 axis to block monocyte recruitment, or modulating macrophage polarization to inhibit the transition to pro-inflammatory M1 phenotype [32,33].

This study found that GLTL can significantly downregulate the expression level of IGF1R total protein, while its main active ingredients, luteolin and naringenin, have limited effects on total protein levels when acting alone, but can significantly inhibit IGF1R phosphorylation. This difference suggests that the formula may regulate IGF1R at multiple levels, including transcriptional control and post-translational modification. The level of IGF1R protein is affected by various post-translation regulatory mechanisms, such as ubiquitin-proteasome-mediated degradation and endosome-lysosomal sorting. Multiple active ingredients in the prescription may act on these different regulatory nodes at the same time, so as to achieve “multi-component, multi-target” synergistic effect. Notably, previous studies have shown that a variety of herbal medicine prescriptions can achieve posttranscriptional regulation of receptors by regulating other axes—for example, Fufang Longdan mixture can directly inhibit IGF1R by upregulating miR-29a-5p to regulate metabolic reprogramming, while Guiqi Baizhu recipe and its active ingredients can inhibit cell proliferation and migration and induce apoptosis by inhibiting IGF1R/PI3K/PDK1 signal axis [34,35]. Based on the above literature background, it is speculated that multiple components in GLTL formula can synergistically act on different regulatory nodes, achieving multi-level inhibition of IGF1R. This multi-level regulatory mechanism not only blocks downstream kinase phosphorylation cascade reactions, but also limits the availability of functional receptors from the source, thereby helping to prevent signal reactivation and providing mechanistic insights for the long-term efficacy of herbal medicine formulas in chronic complex diseases.

Finally, molecular docking screening results showed that all 10 natural compounds in the GLTL formula exhibited certain binding potential to IGF1R, with the binding activity of luteolin and naringenin being the most significant, and their binding stability to IGF1R was further confirmed by MD simulations. It is worth noting that previous computational studies have suggested that luteolin may bind to IGF1R and inhibit its ligand IGF1 binding, but there have been no pharmacological reports in this model [36]. Naringenin, as a structurally similar flavonoid, had its IGF1R-targeting activity systematically validated for the first time in this study. These computational predictions were mutually corroborated by the preceding CETSA and DARTS results, collectively indicating that, among the constituents screened, luteolin and naringenin show the strongest IGF1R engagement and therefore serve as representative IGF1R-targeting constituents of the formula; because the DARTS assay was performed in the whole-cell format, these data should be regarded as evidence of intracellular target engagement rather than as stand-alone proof of direct binding. Moreover, although the remaining eight compounds were not further validated in the present study, some of them, such as Apigenin and Quercetin, may also possess certain binding potential with IGF1R, and the possibility that they may participate in the regulation of IGF1R signaling through synergistic mechanisms still awaits further investigation.

## 4. Materials and Methods

### 4.1. Materials and Chemicals

The herbal medicines used in this study include Platycodon grandiflorum, Moutan Cortex, and Glycyrrhiza uralensis (supplied by Chengdu Xinfuyuan Traditional Chinese Medicine Decoction Pieces Co., Ltd., Chengdu, China, with batch numbers 25050103, 24080102, and 24080201, respectively); Cinnamomum cassia, Trichosanthes kirilowii, and Radix Paeoniae Rubra (supplied by Sichuan Guoqiang Traditional Chinese Medicine Decoction Pieces Co., Ltd., Chengdu, China, with batch numbers 2408074, 2407242, and 2408095, respectively); Poria cocos, Ephedra sinica, Allium macrostemon, and Ardisia japonica (supplied by Chengdu Ji’an Kang Pharmaceutical Co., Ltd., Chengdu, China, with batch numbers 250626-72, 240416-61, 240816-51, and 240419-62, respectively); and Persicae Semen (supplied by Chengdu Kangmei Pharmaceutical Production Co., Ltd., Chengdu, China, with batch number 250500861). Luteolin (Shanghai Standard Technology, Shanghai, China, 13537-G260302); Naringenin (Shanghai Standard Technology, Shanghai, China, 16647-G260301); Linsitinib (MCE, HY-10191).

Dulbecco’s Modified Eagle Medium (DMEM) F-12 (CORNING, 10-092-CV), fetal bovine serum (FBS) (CORNING, 35-081-CV), Platelet-Derived Growth Factor BB (PDGF-BB, MCE, HY-P7278), CCK-8 (Zeheng Biotech, China, ZH0025), E-Click EdU-594 Cell Proliferation Detection Kit (Elabscience, Wuhan, China, E-CK-A377), Annexin V-FITC/PI Apoptosis Detection Kit (4A Biotech, Beijing, China, FXP018), polymerase chain reaction (PCR) test kit (EXONGEN, Chengdu, China, A504-02). Antibodies against IGF1 (ab106836), IGF1R (ab182408) and p-IGF1R (ab314116) were obtained from Abcam (Cambridge, UK). The PCNA (AC058), BCL2 (A19693), Cyclin D1 (A11022), α-SMA (A7248), Ki-67 (A23722), active-caspase3 (A11021) antibodies were obtained from ABclonal (Wuhan, China). Antibodies for PI3K (T40115), P-PI3K (T40116), AKT (T55561), P-AKT (T40067), and β-actin (P30002) were from Abmart (Shanghai, China). Pronase E (MCE, HY-114158).

### 4.2. Analytical Instrumentation

The high-resolution mass spectrometry analysis was performed using a Q Exactive™ Quadrupole-Orbitrap™ mass spectrometer (Thermo Fisher Scientific, Waltham, MA, USA), equipped with a Thermo Scientific Accucore™ C18 column (3 × 100 mm, 2.6 μm). Cellular microscopic observation was conducted using an IX83 microscope (Olympus Corporation, Tokyo, Japan). Confocal images were acquired on a TCS SP8 STED confocal microscope (Leica Microsystems, Wetzlar, Germany). The gel imaging was performed using a UVP ChemStudio system, and quantitative real-time PCR was performed using a qTOWER^3^G instrument (both from Analytik Jena, Jena, Germany). Flow cytometry analysis was performed on a CytoFLEX LX flow cytometer (Beckman Coulter, Brea, CA, USA).

### 4.3. Data Sources and Retrieval

Pulmonary hypertension (PH)-related datasets were retrieved from the Gene Expression Omnibus (GEO) database (https://www.ncbi.nlm.nih.gov/geo; accessed on 17 September 2025), and array-based expression profiles of Homo sapiens were selected for analysis. The datasets GSE24988, GSE113439, and GSE117261 were selected as the bulk RNA-seq training sets for PH and control samples, all the testing samples were derived from lung tissues. Because these datasets comprise heterogeneous pulmonary hypertension groups (including PH associated with pulmonary fibrosis in GSE24988), sample-level PH subgroup definitions are provided in Table S4, and the bioinformatic findings should therefore be interpreted as convergent vascular-remodeling signatures shared across PH groups rather than Group 1 PAH-specific changes. Throughout the bioinformatic analyses, the pooled disease samples are referred to as the disease group. The dataset GSE15197 was subsequently used as an independent validation set for evaluating the robustness of target-gene expression trends. For scRNA-seq data, datasets were also obtained from the GEO database by specifying the experiment type as “Expression profiling by high throughput sequencing” and the organism as “Homo sapiens”. The dataset GSE228644 was ultimately selected for downstream 10X scRNA-seq analysis. This dataset consisted of “Medium/small-size pulmonary artery” tissue samples, with cell types annotated as “PA resident cells”, and included three PAH patients (GSM6424658, GSM6424659, GSM6424660). The detailed information is summarized in Table S4.

### 4.4. Gene Acquisition

The collected training sets served as the source for disease-related gene screening. First, probe annotation files from the same platform were used to map gene IDs, avoiding cross-platform probe discrepancies. To minimize inter-sample variability, the ComBat. function from the R package sva was employed to remove batch effects during dataset integration. Differential expression analysis between the disease group and controls (N) was performed using the functions in limma package. The logFC value was calculated to quantify intergroup gene expression differences. The differential expression threshold was set to *p* < 0.05 and |log2FC| ≥ 0.4 to identify DEGs.

We also utilized WGCNA to identify gene sets highly associated with the disease from gene expression profiles, leveraging its ability to reveal inter-gene relationships and gene-phenotype associations. Initially, outliers were detected and removed using the goodSamplesGenes function of the WGCNA package. The pickSoftThreshold function was employed to select the soft-thresholding power needed for subsequent network construction. After dimensionality reduction of genes into multiple modules, module-trait relationships were calculated to derive gene significance and module membership metrics. In the final step, the findings from WGCNA and DEG analyses were combined, duplicates removed, yielding a consolidated set of disease-related genes for downstream analyses.

### 4.5. Collection of GLTL Components and Their Corresponding Target Genes

The components of the GLTL formula were accurately weighed, immersed in 1400 mL of purified water for 30 min, and then refluxed for 60 min. The filtrate was collected and concentrated to a concentration of 0.5g/mL. The supernatant was passed through a 0.22 μm microporous filter, and the resulting filtrate was employed as the test solution. The compounds in the GLTL formulation were analyzed using ultra-performance liquid chromatography (UPLC)-MS/MS; MS/MS fragments were matched against the mzCloud library in Compound Discoverer (v3.3), with a mass accuracy threshold of 5 ppm (Tables S5 and S6). The excess filtrate was pre-frozen and then vacuum-dried at −50 °C for 24 h to yield a freeze-dried powder (yield: 15.6%); the freeze-dried powder was dissolved in PBS to a concentration of 1 mg/mL to prepare a stock solution, which was then used in subsequent dosing experiments. Subsequently, the components detected by LC-MS were combined and analyzed with the components in the blood component database. The blood-absorbed components of 11 Chinese herbal medicines in the GLTL were collected from the DCABM-TCM (https://ngdc.cncb.ac.cn/databasecommons/database/id/8439, accessed on 20 September 2025) and the previous literature. Additionally, compounds meeting the criteria of oral bioavailability (OB) ≥ 30% and drug-likeness (DL) ≥ 0.18 in the Traditional Chinese Medicine Systems Pharmacology (TCMSP) database (https://tcmsp-e.com/index.php; accessed on 22 September 2025) served as supplementary ingredients. The Simplified Molecular Input Line Entry System (SMILES) strings of these compounds were retrieved from the PubChem database (https://pubchem.ncbi.nlm.nih.gov/, accessed on 22 September 2025) and subsequently applied for target prediction using SwissTargetPrediction (https://www.swisstargetprediction.ch/index.php; accessed on 22 September 2025). Genes with a “Probability” value greater than zero were selected as candidate targets. Further supplementation was performed using target information from the DrugBank (https://go.drugbank.com/; accessed on 22 September 2025), TCMIP, and Herb databases (http://herb.ac.cn/; accessed on 22 September 2025) to comprehensively identify target genes of the herbal components. The obtained drug-related genes will be intersected with the previously screened disease-related genes, and the obtained genes will be the key gene set. The resulting overlapping genes were used to construct an interactive “Herb-Compound-Disease-Target” network.

### 4.6. Acquisition of Key Genes and Gene Enrichment Analysis

Disease-associated and drug-related genes were combined, then submitted to the STRING database (https://cn.string-db.org/; accessed on 23 September 2025) under the “Homo sapiens” setting for analysis. The parameters “hide disconnected nodes in the network” and “highest confidence (0.900)” were applied, and the resulting data were exported in a format compatible with Cytoscape (v3.10.2). Using the “Analyze Network” function in Cytoscape, various parameters were calculated. Initially, genes whose degree values were more than twice the median were retained for subsequent analysis. Second, the CytoHubba plugin was used to analyze genes based on MCC and MNC centrality. The genes identified from both analyses were combined. Next, the MCODE plugin was applied with default parameters to identify the two highest-scoring clusters. Finally, the intersection between the genes identified by the three aforementioned approaches and the key gene set was taken to define the hub genes. To clarify the biological significance of the identified key genes, GO and KEGG enrichment analyses were performed. The Wei-Sheng-Xin platform (https://www.bioinformatics.com.cn/; accessed on 23 September 2025) was utilized for GO and KEGG enrichment analyses. Pathways with *p* < 0.05 were deemed significant, and several of the most relevant pathways were visualized.

### 4.7. Immune Infiltration Analysis

By analyzing immune cell infiltration, both the evaluation of cellular presence in affected regions and the recognition of principal immune subtypes relevant to pulmonary hypertension can be achieved. This analysis was performed using the Cell-type Identification by Estimating Relative Subsets of RNA Transcripts (CIBERSORT) algorithm. First, the expression matrix was standardized, and the LM22 signature matrix was used as a reference for cell type-specific gene expression profiles. Immune cell abundance was estimated using the above algorithm with default parameters, 5000 permutations, and the standardization option enabled to ensure result reliability. To comprehensively visualize the distribution characteristics of immune cells in the dataset, a stacking ratio map was drawn. In addition, immune cell abundance data were merged with sample grouping information to illustrate their relationship. A correlation heatmap was generated using the corrplot package to display these relationships. Boxplots were generated using the ggplot2 package to compare immune cell abundance between groups. Finally, to investigate the correlation between key gene expression and immune cells, the gene expression data were merged with the transposed immune cell abundance data, Pearson correlation coefficients were calculated to construct a correlation matrix, and the correlation between genes and immune cells was visualized.

### 4.8. scRNA-seq Data Analysis

After obtaining the PAH-related scRNA-seq data, a Seurat object was created using the CreateSeuratObject function. Quality control filtering was performed according to the following criteria: cells were retained if they expressed 200–10,000 genes (nFeature_RNA), had at least 1000 detected molecules (nCount_RNA), and showed mitochondrial and ribosomal gene proportions of ≤20% each. Subsequently, data were normalized using the LogNormalize method, and highly variable genes (HVGs) were identified with the FindVariableFeatures function; the top 2000 HVGs were selected for downstream analysis. Prior to dimensionality reduction, scaling was applied to ensure equal weight of expression levels across all genes. Subsequently, PCA was performed and the optimal principal components were selected for dimensionality reduction. Using the dimension-reduced data, a cell adjacency network was constructed with the FindNeighbors function, followed by cell clustering with the FindClusters function. To determine the optimal clustering resolution, a parameter range of 0.2 to 1.2 (step size 0.1) was tested, and a resolution of 0.8 was ultimately selected for subsequent analysis. To visualize the data, non-linear dimensionality reduction was performed using RunUMAP and RunTSNE functions, generating UMAP and t-SNE scatter plots, respectively, to visually display the cell population structure. Considering the potential bias in automatic annotation, we used marker genes from the CellMarker database and related literature for artificial cell type annotation. The annotated cell subtypes were visualized and their proportions in each sample were calculated. CellChat was used to analyze intercellular communication networks and to reveal interaction patterns among cell populations. The Monocle package was used for pseudotime analysis to infer developmental trajectories and to depict dynamic transitions between cell states. Subsequently, the expression patterns of key gene sets across cell types were evaluated using AUCell and AddModuleScore. The AUCell package was used to calculate and visualize gene set activity scores in individual cells, and the AddModuleScore function in Seurat was used to verify the enrichment of key gene sets in specific cell populations, thereby improving the reliability of the scoring results.

### 4.9. Integrated Analysis of Disease Association, Single-Gene Enrichment, and Pathway Activity Assessment

A comprehensive analysis framework was used to systematically evaluate the association of key genes with disease status and the functional correlation of target genes. A multivariable logistic regression model was constructed to explore the association of the key genes, and a nomogram was drawn to visualize the contribution of each gene. The discriminative ability of the joint model and individual genes was evaluated using the ROC curve as the evaluation metric. Additionally, DCA was performed, assessing the model’s net benefit across various threshold probabilities over specific threshold intervals. For further validation of the screened targets, ssGSEA and GSVA were carried out with GMT-format annotation files retrieved from the GSEA official website (https://www.gsea-msigdb.org/gsea/msigdb; accessed on 13 October 2025). By combining the expression trends of corresponding genes obtained from multiple datasets collected from the GEO database, the screened genes were jointly validated. To explore the association between the target gene IGF1R and biological functions, a Spearman correlation test was used to quantify the correlation between IGF1R and all other genes. Subsequently, enrichment analysis was performed via the GSEA function, and the significantly associated functional annotations were visualized. Finally, the coordinated activation status of the IGF1R/PI3K/AKT pathway at the single sample level was further investigated through GSVA and ssGSEA. The pathway activity scores and trends obtained from these two methods reflect the overall activation status of this pathway at the sample level in both the training and validation datasets.

### 4.10. Molecular Docking and Molecular Dynamics Simulation

The IGF1R protein structure was downloaded from the RCSB Protein Data Bank (PDB) (https://www.rcsb.org/; accessed on 20 October 2025); PDB ID: 1P4O. Based on PPI network analysis, structural files of candidate small molecules were retrieved from PubChem for docking, and both the protein and small-molecule ligands were preprocessed. Molecular structures underwent energy minimization with ChemDraw 3D (v20.0.0), while the protein was prepared by removing water molecules. The binding pocket of IGF1R (center: X = 20, Y = 72, Z = 28) was determined with CB-DOCK2 (http://cao.labshare.cn:10380/cb-dock2/php/blinddock.php; accessed on 27 October 2025). Docking was subsequently carried out using AutoDock (v4.2.6), and PyMOL (v 2.2.3) was employed to visualize the results. Because PDB 1P4O contains no co-crystallized ligand, reference-ligand re-docking was not performed; docking results were used to rank the candidates rather than to estimate binding affinities. MD simulations of the complexes were run with Groningen Machine for Chemical Simulations (GROMACS) 2022.2. Protein topologies were built using Amber99SB-ILDN, and ligand topologies with RESP charges were prepared via GAFF2. Systems were solvated in cubic boxes of TIP3P water, with the protein at least 1.0 nm from the box boundary. Na^+^ and Cl^−^ ions were added to obtain a 0.15 M physiological salt concentration. Long-range electrostatics were handled by PME; cutoffs for van der Waals and short-range electrostatics were both 1.2 nm. Bonds were constrained via LINCS, and a 2fs integration step was used. Prior to the production run, energy minimization was performed using the steepest descent and conjugate gradient methods, followed by 1 ns NVT and 1 ns NPT equilibration. During the equilibration stages, positional restraints were applied to both the protein and the ligand. The temperature was maintained at 300 K using a V-rescale thermostat and the pressure at 1 bar using a Parrinello-Rahman barostat. After equilibration, a 100 ns unrestrained production simulation was performed for each system at 300 K and 1 bar. Trajectory analysis was then performed using GROMACS built-in tools.

### 4.11. Cell Extraction and Cultivation

Primary PASMCs were isolated from Sprague-Dawley rats. Six specific-pathogen-free (SPF) grade SD male rats were anesthetized with tribromoethanol (20 μL/g), euthanized by bleeding from the abdominal aorta, and their heart and lung tissues were collected. Next, the pulmonary arterial tissue was sectioned into 1-mm^2^ fragments, which were uniformly placed into culture dishes and incubated in DMEM/F-12 with 20% FBS and 1% antibiotic mixture (penicillin/streptomycin). For subsequent assays, passage-3 to -8 cells were seeded in DMEM/F-12 (10% FBS, 1% P/S) and kept at 37 °C under 5% CO_2_. Once the cells attained roughly 70% confluency, they were assigned to either a control condition (serum-free medium with antibiotics) or a model condition (exposure to 20 ng/mL PDGF-BB; serum-free medium with antibiotics) [37]. Low-dose group (GLTL-L): PASMCs were treated simultaneously with PDGF-BB and 100 μg/mL GLTL. Medium-dose group (GLTL-M): PASMCs were treated simultaneously with PDGF-BB and 200 μg/mL GLTL. High-dose group (GLTL-H): PASMCs were treated simultaneously with PDGF-BB and 400 μg/mL GLTL. Luteolin group (Lut): PASMCs were treated simultaneously with PDGF-BB and 70 μM luteolin. Naringenin group (Nar): PASMCs were treated simultaneously with PDGF-BB and 90 μM naringenin. Linsitinib group (Linsitinib): PASMCs were treated with both PDGF-BB and 3.51 μM linsitinib. The concentration of PDGF-BB for model establishment and the dosage of GLTL or compounds for administration were both screened through experiments (see Section 2.1 and Section 2.9 for details). All treatments were conducted for 24 h.

### 4.12. Cell Viability Assay

PASMCs were seeded into 96-well plates and cultured for 24 h. Following the assigned group-based interventions, the cells underwent an additional 24 h of cultivation. Next, each well received 10% CCK-8 reagent, followed by a 60 min incubation at 37 °C. Thereafter, the optical density per well was determined at 450 nm by means of a microplate reader.

### 4.13. Scratch Wound Healing Test

PASMCs were seeded in six-well plates and cultured until the monolayer reached 80% coverage. Then, a sterile 200 µL pipette tip was drawn across the cell layer to make straight wounds. Following the scratch, the cells were subjected to different treatments according to the experimental groups. To assess migratory ability, wound closure was observed and imaged under the microscope at 0 and after 24 h.

### 4.14. Flow Cytometry

PASMCs harvested during exponential growth were seeded into six-well plates and incubated until 70% confluence. Subsequently, according to the experimental design, the cells were exposed to PDGF-BB or related test substance for 24 h. After treatment, the culture supernatant was collected and the adherent cells were digested with ethylenediaminetetraacetic acid (EDTA)-free trypsin. The digested cells were then mixed again with the previously retained supernatant. The resulting cell suspension was centrifuged at 1000 rpm for 5 min, and the pellet was washed twice with pre-cooled phosphate-buffered saline (PBS). The cells were then stained with the Annexin V-FITC apoptosis detection kit according to the manufacturer’s instructions. Fluorescence was read at 525 nm (FITC) and 575 nm (PI) using the designated channels, and the apoptotic rate was calculated using the appropriate analysis software.

### 4.15. EdU Staining

The proliferative capacity of PASMCs was assessed using the E-Click EdU-594 Cell Proliferation Detection Kit (Elabscience, Wuhan, China). After treatment with 20 ng/mL PDGF-BB or medication for 24 h, each group of cells was co-incubated with 50 μM EdU for 2 h for labeling. After the supernatant was aspirated, the adherent cells underwent fixation with 4% paraformaldehyde (PFA) (15 min, room temperature). After washing, cells underwent a 20-min permeabilization step. After a 30 min dark incubation with the Click reagent at 25 °C, the cells were treated with 4′,6-diamidino-2-phenylindole (DAPI) for 10 min to label nuclei, and subsequently imaged under a fluorescent microscope.

### 4.16. Immunofluorescence

Cells grown on confocal dishes were subjected to fixation in 4% paraformaldehyde and then permeabilization using 0.5% Triton X-100 (BioFroxx, Germany, 1139ML100). Thereafter, the specimens were treated with 5% bovine serum albumin (BSA) for 60 min at ambient temperature to block nonspecific binding, followed by an overnight incubation at 4 °C with appropriate primary antibodies (α-SMA, Calponin and Ki-67). After rinsing, the samples received species-specific fluorescent secondary antibodies and were kept in the dark for 1 h at room temperature. The dishes were then washed three times, nuclei were counterstained with DAPI, and the samples were immediately examined under a confocal laser scanning microscope.

### 4.17. Western Blotting Assay

The collected cells were lysed in radioimmunoprecipitation assay (RIPA) lysis buffer for half an hour. After centrifugation, the supernatant was retained, and the protein content of PASMC lysate was quantified using bicinchoninic acid (BCA) method and adjusted to an equal concentration. The samples were mixed with loading buffer and heated at 100 °C for 10 min to denature the proteins. The protein separated by electrophoresis was transferred onto a polyvinylidene difluoride (PVDF) membrane and then blocked with Tris-buffered saline with Tween 20 (TBST) containing 5% BSA at room temperature for 1 h. After a wash step, the blots were incubated overnight at 4 °C with primary antibodies directed against IGF1, IGF1R, p-IGF1R, PI3K, p-PI3K, AKT, p-AKT, BCL-2, Cyclin D1, PCNA and Active-caspase3. Then, the membranes were exposed to secondary antibodies for 60 min, followed by chemiluminescent detection. The resultant bands were quantified by densitometry with ImageJ (v1.54p) software.

### 4.18. qRT-PCR

Total RNA was isolated from PASMC cultures employing a commercial extraction kit, then reverse-transcribed to cDNA under the conditions of 25 °C for 5 min, 55 °C for 10 min, and 85 °C for 5 s. For real-time PCR analysis, the cDNA was amplified with a protocol consisting of 95 °C for 3 min, 40 cycles of 95 °C for 5 s and 60 °C for 30 s, and a melting-curve stage. Primer sequences are detailed in Supplementary Table S7.

### 4.19. CETSA and DARTS for Evaluating Drug-Target Interactions

For the cellular thermal shift assay (CETSA) procedure, PASMCs were incubated with the candidate substances (Lut:70 μM; Nar:90 μM) for 2 h, then gathered and rinsed with protease-inhibitor-containing PBS. After being re-suspended in the same buffer, the cells were dispensed into PCR tubes and exposed to a stepped temperature ramp (37, 42, 47, 52, 57, 62, 67 °C) in a thermal cycler for 5 min, followed by cooling at room temperature for 3 min and subsequent chilling on ice. Subsequently, the cells were rapidly subjected to three freeze–thaw cycles in liquid nitrogen to achieve lysis. After centrifugation of the lysate (13,000 rpm, 10 min, 4 °C), the clear supernatant was collected, mixed with loading buffer, and boiled at 100 °C for 10 min to denature the proteins; the residual IGF1R signal was then detected by Western blotting.

In the drug affinity responsive target stability (DARTS) experiment, a whole-cell (live-cell pretreatment) variant of the assay was used: intact PASMCs were incubated with the test compounds for 2 h so that target engagement occurred under native cellular conditions (native receptor conformation, membrane context and endogenous ligands), consistent with the conditions of the CETSA experiments. The supernatant was collected after centrifugation and protein levels were normalized using the BCA quantification method. Subsequently, these lysates were incubated with Pronase E (1:200, 1:400, 1:800, and 1:1600) at 4 °C for 30 min. The proteolytic reaction was terminated by heating the samples at 100 °C for 10 min. Finally, the abundance of the complete target protein was determined by Western blot analysis. Because no protease-free control lane was included, β-actin at the lowest protease concentration (1:1600) was used as the internal loading reference.

### 4.20. Statistical Analysis

Experimental data are expressed as the mean ± standard deviation (SD). Statistical analyses were performed using GraphPad Prism (version 9.5.1). Comparisons between two groups were made using an unpaired *t*-test, and comparisons among multiple groups were made using one-way analysis of variance (ANOVA) followed by Dunnett’s post hoc test against the model group. Homogeneity of variance was assessed using the Brown–Forsythe test. *p* < 0.05 was considered statistically significant. *n* = 3 biologically independent replicates, i.e., primary cells isolated from three individual rats, each dissociated, cultured and treated independently.

## 5. Conclusions

In summary, luteolin and naringenin have been identified as potential active ingredients that inhibit PDGF-BB-induced PASMC proliferation in vitro. Both compounds engaged IGF1R and suppressed IGF-1/IGF1R signaling, thereby reducing PI3K/AKT phosphorylation and the downstream expression of Cyclin D1, BCL2, PCNA and Ki67, which in turn suppressed the excessive proliferation and promoted the apoptosis of PDGF-BB-stimulated PASMCs. These findings provide a useful reference for screening active constituents against PAH from natural products.

## 6. Limitations

This study has several limitations. First, all experiments were conducted in primary PASMCs isolated from SD rats without in vivo validation; therefore, all conclusions are restricted to the in vitro setting, and the functional relevance of the IGF1R/PI3K/AKT pathway remains to be confirmed in animal models of pulmonary arterial hypertension. Second, the chemical composition of GLTL was characterized by UHPLC-Q Exactive MS, which defines its constituents but does not reflect their in vivo fate, including absorption, distribution, metabolism, and bioavailability; pharmacokinetic and tissue distribution studies based on LC-MS/MS are therefore required [38]. Third, the isolated compounds were used at half of their own IC_50_ values, at which the CCK-8 signal of unstimulated PASMCs was reduced by approximately 30%. Because CCK-8 reports overall metabolic activity and cannot separate pathway-specific cytostasis from non-specific cytotoxicity, the mechanistic conclusion rests on the pathway-level concordance with linsitinib (loss of p-IGF1R and p-AKT without a change in total IGF1R) and on proliferation- and apoptosis-specific readouts (EdU, Ki67, Annexin V/PI and active caspase-3), rather than on the viability assay alone. These concentrations also exceed those reported in plasma after oral administration, where luteolin reaches approximately 3 μg/mL in rats [39] and naringenin requires sensitive LC-MS/MS quantification after oral dosing [40]; the isolated-compound experiments are therefore best interpreted as mechanistic characterization of representative constituents rather than as a model of systemic exposure [41]. Ephedrine-like alkaloids (e.g., ephedrine and pseudoephedrine) were detected in GLTL (Table 1). Although sympathomimetic amines have been implicated in drug-induced PAH, our RCT [14] in COPD-associated PAH showed no sympathomimetic adverse events or worsening of pulmonary hemodynamics. However, alkaloid exposure and long-term cardiovascular safety remain uncharacterized, especially in Group 1 PAH. Therefore, ephedra-containing preparations should be used cautiously in PAH patients, and dedicated pharmacovigilance and dose-optimization studies are needed. The scRNA-seq analysis included only three PAH samples without healthy controls. Thus, pseudotime trajectories and enrichment scores are presented as descriptive characterization of pathway activity within PAH tissue, not disease-specific differentiation or activation; healthy-control datasets are needed to establish PAH specificity. These findings are therefore hypothesis-generating and interpreted in conjunction with the bulk and in vitro data.

## Figures and Tables

**Table 1:** Identification of chemical constituents in the GLTL formulation using UHPLC-MS/MS.

Name	Formula	Molecular Weight	Actual Value (*m*/*z*)	Theoretical Value (*m*/*z*)	PPM	RT (min)	Pattern
Ephedrine	C_10_H_15_NO	165.11532	166.1226	166.12264	−0.24	6.087	[M + H]
Pseudoephedrine	C_10_H_15_NO	165.11531	166.12259	166.12264	−0.31	6.817	[M + H]
N-Methylephedrine	C_11_H_17_NO	179.13105	180.13834	180.13829	0.27	7.758	[M + H]
Albiflorin	C_23_H_28_O_11_	480.16275	481.16986	481.17044	−1.20	16.859	[M + H]
Malic acid	C_4_H_6_O_5_	134.02073	133.01446	133.01425	1.60	1.465	[M − H]
Paeoniflorin	C_23_H_28_O_11_	480.16313	479.15598	479.15588	0.20	18.468	[M − H]
Bergenin	C_14_H_16_O_9_	328.07926	329.0863	329.08671	−1.24	10.456	[M + H]
4-Methoxycinnamic acid	C_10_H_10_O_3_	178.06286	179.07019	179.07027	−0.45	18.483	[M + H]
DL-Arginine	C_6_H_14_N_4_O_2_	174.11156	175.11884	175.11895	−0.64	1.391	[M + H]
Proline	C_5_H_9_NO_2_	115.06333	134.08112	134.08117	−0.37	1.442	[M + H_3_O]
Isoliquiritigenin	C_15_H_12_O_4_	256.07314	257.08041	257.08084	−1.65	20.835	[M + H]
L-Phenylalanine	C_9_H_11_NO_2_	165.079	166.08627	166.08626	0.09	3.798	[M + H]
DL-Norleucine	C_6_H_13_NO_2_	131.09468	132.10196	132.10191	0.41	2.258	[M + H]
Glycyrrhizic acid	C_42_H_62_O_16_	822.40332	821.3963	821.39785	−1.88	29.369	[M − H]
Adenosine	C_10_H_13_N_5_O_4_	267.09654	268.10382	268.10403	−0.78	2.263	[M + H]
L-Pyroglutamic acid	C_5_H_7_NO_3_	129.04269	130.04996	130.04987	0.70	1.835	[M + H]
Gallic acid	C_7_H_6_O_5_	170.02095	169.01366	169.01425	−3.47	2.544	[M − H]
18-β-Glycyrrhetinic acid	C_30_H_46_O_4_	470.33899	471.34634	471.34689	−1.16	29.38	[M + H]
Liquiritin	C_21_H_22_O_9_	418.1262	417.11899	417.11911	−0.28	20.811	[M − H]
Oxypaeoniflorin	C_23_H_28_O_12_	496.1582	495.15091	495.1508	0.22	12.154	[M − H]
Sucrose	C_12_H_22_O_11_	342.11628	341.10892	341.10893	−0.04	1.368	[M − H]
Benzoylpaeoniflorin	C_30_H_32_O_12_	584.18938	583.18256	583.1821	0.79	26.934	[M − H]
Licuraside	C_26_H_30_O_13_	550.16845	549.16119	549.16136	−0.32	20.984	[M − H]
Hordenine	C_10_H_15_NO	165.11534	166.12262	166.12264	−0.12	2.786	[M + H]
Cathine	C_9_H_13_NO	151.09963	152.10696	152.10699	−0.20	5.272	[M + H]
liquiritigenin	C_15_H_12_O_4_	256.07314	257.08041	257.08084	−1.65	21.018	[M + H]
Apocynin	C_9_H_10_O_3_	166.0629	167.07018	167.07027	−0.54	16.34	[M + H]
Trigonelline	C_7_H_7_NO_2_	137.04762	138.0549	138.05496	−0.40	1.418	[M + H]
Paeonol	C_9_H_10_O_3_	166.0629	167.07025	167.07027	−0.12	17.526	[M + H]
Tyrosine	C_9_H_11_NO_3_	181.07388	182.08118	182.08117	0.06	1.983	[M + H]
Valine	C_5_H_11_NO_2_	117.07918	118.08648	118.08626	1.90	1.549	[M + H]
Pyrogallol	C_6_H_6_O_3_	126.03089	125.02461	125.02442	1.54	2.737	[M − H]
Catechin	C_15_H_14_O_6_	290.0791	289.0719	289.07176	0.48	11.271	[M − H]
Licoricesaponin G_2_	C_42_H_62_O_17_	838.39788	837.3913	837.39142	−0.15	28.484	[M − H]
Neoliquiritin	C_21_H_22_O_9_	418.12627	417.11902	417.11911	−0.20	20.648	[M − H]
Guanosine	C_10_H_13_N_5_O_5_	283.09145	284.09872	284.09895	−0.79	2.578	[M + H]
Quercitrin	C_21_H_20_O_11_	448.10045	447.09335	447.09328	0.15	22.535	[M − H]
Cinnamic acid	C_9_H_8_O_2_	148.05237	149.05974	149.05971	0.23	3.602	[M + H]
Cinnamaldehyde	C_9_H_8_O	132.05746	133.06468	133.06479	−0.84	23.358	[M + H]
Ellagic acid	C_14_H_6_O_8_	302.00618	300.9989	300.99899	−0.30	20.766	[M − H]
Eucalyptol	C_10_H_18_O	154.13581	155.14304	155.14304	−0.01	18.013	[M + H]
Licoricesaponin H_2_	C_42_H_62_O_16_	822.40326	821.39667	821.39785	−1.43	30.294	[M − H]
Norephedrine	C_9_ H_13_NO	151.09963	152.10696	152.10699	−0.20	4.884	[M + H]
Phenylacetone	C_9_ H_10_O	134.07312	135.08046	135.08044	0.14	6.141	[M + H]
18-α-Glycyrrhetinic acid	C_30_H_46_O_4_	470.33899	471.34547	471.34689	−3.01	30.294	[M + H]
Protocatechualdehyde	C_7_H_6_O_3_	138.03091	137.02467	137.02442	1.84	21.752	[M − H]
Acetophenone	C_8_H_8_O	120.0577	121.06493	121.06479	1.14	9.07	[M + H]
Schaftoside	C_26_H_28_O_14_	564.14763	565.15479	565.15518	−0.69	19.674	[M + H]
5-Hydroxymethylfurfural	C_6_H_6_O_3_	126.03186	127.03912	127.03897	1.18	1.613	[M + H]
Nicotinamide	C_6_H_6_N_2_O	122.04821	123.05546	123.05529	1.39	1.774	[M + H]
Nicotinic acid	C_6_ H_5_NO_2_	123.0322	124.0395	124.03931	1.57	1.711	[M + H]
Loliolide	C_11_H_16_O_3_	196.10976	197.11703	197.11722	−0.97	20.755	[M + H]
Ononin	C_22_H_22_O_9_	430.12587	431.13315	431.13366	−1.18	24.251	[M + H]
Epicatechin	C_15_H_14_O_6_	290.07907	289.07193	289.07176	0.58	16.29	[M − H]
Quercetin	C_15_H_10_O_7_	302.04222	303.0495	303.04993	−1.42	22.522	[M + H]
Safflomin A	C_27_H_32_O_16_	612.16878	611.1615	611.16176	−0.42	21.045	[M − H]
Licoagroside B	C_18_H_24_O_12_	432.12649	433.13373	433.13405	−0.74	15.281	[M + H]
2-Isopropylmalic acid	C_7_H_12_O_5_	176.06793	175.06058	175.0612	−3.52	9.155	[M − H]
Licoricesaponin A_3_	C_48_H_72_O_21_	984.45651	985.46313	985.46389	−0.77	26.637	[M + H]
Vicenin-2	C_27_H_30_O_15_	594.15847	595.16559	595.16575	−0.26	18.334	[M + H]
Paeonolide	C_20_H_28_O_12_	460.15827	459.151	459.1508	0.44	17.527	[M − H]
Citric acid	C_6_H_8_O_7_	192.02703	193.03442	193.03428	0.73	1.745	[M + H]
Citramalic acid	C_5_H_8_O_5_	148.0365	147.02922	147.0299	−4.60	2.115	[M − H]
Pinocembrin	C_15_H_12_O_4_	256.07329	257.08057	257.08084	−1.03	20.601	[M + H]
Isoliquiritin	C_21_H_22_O_9_	418.12624	417.11932	417.11911	0.51	24.132	[M − H]
Benzoic acid	C_7_H_6_O_2_	122.03697	123.04424	123.04406	1.50	20.836	[M + H]
Cordycepin	C_10_H_13_N_5_O_3_	251.10169	252.10899	252.10912	−0.50	2.439	[M + H]
Procyanidin B2	C_30_H_26_O_12_	578.14247	579.14954	579.1497	−0.28	10.06	[M + H]
Gentisic acid	C_7_H_6_O_4_	154.02594	153.01866	153.01933	−4.39	5.219	[M − H]
1,2,3,6-Tetra-O-galloyl-β-D-glucose	C_34_H_28_O_22_	788.10744	787.10016	787.09995	0.27	21.567	[M − H]
Afzelin	C_21_H_20_O_10_	432.10561	431.09848	431.09837	0.26	23.721	[M − H]
2-Hydroxycinnamic acid	C_9_H_8_O_3_	164.04732	165.0546	165.05462	−0.13	2.005	[M + H]
Kaempferol	C_15_H_10_O_6_	286.0475	287.05478	287.05501	−0.82	23.722	[M + H]
Caffeic acid	C_9_H_8_O_4_	180.04173	179.03445	179.03498	−2.97	12.172	[M − H]
Guanine	C_5_H_5_N_5_O	151.04939	152.05669	152.05669	0.02	2.427	[M + H]
Procyanidin B1	C_30_H_26_O_12_	578.14246	579.14972	579.1497	0.03	14.715	[M + H]
p-cymene	C_10_H_14_	134.1095	135.11678	135.11683	−0.35	20.662	[M + H]
Morin	C_15_H_10_O_7_	302.04222	303.04947	303.04993	−1.51	22.391	[M + H]
Coumarin	C_9_H_6_O_2_	146.03667	147.04395	147.04406	−0.72	22.422	[M + H]
Gallocatechin	C_15_H_14_O_7_	306.07412	305.06693	305.06668	0.83	5.399	[M − H]
lsovitexin	C_21_H_20_O_10_	432.10518	433.11249	433.11292	−1.00	20.925	[M + H]
Licochalcone B	C_16_H_14_O_5_	286.08379	287.091	287.0914	−1.39	23.162	[M + H]
Myricitrin	C_21_H_20_O_12_	464.09548	463.08844	463.0882	0.52	20.837	[M − H]
Epigallocatechin	C_15_H_14_O_7_	306.07405	305.06677	305.06668	0.31	10.46	[M − H]
Naringenin	C_15_H_12_O_5_	272.06804	273.07532	273.07575	−1.57	20.814	[M + H]
Protocatechuic acid	C_7_H_6_O_4_	154.0264	155.03372	155.03389	−1.07	20.834	[M + H]
Naringenin chalcone	C_15_H_12_O_5_	272.06804	273.07553	273.07575	−0.81	20.329	[M + H]
Mandelic acid	C_8_H_8_O_3_	152.04652	151.03943	151.04007	−4.22	20.922	[M − H]
Prunin	C_21_H_22_O_10_	434.12126	433.11398	433.11402	−0.09	22.949	[M − H]
Uridine	C_9_H_12_N_2_O_6_	244.06946	243.06218	243.06226	−0.33	1.959	[M − H]
Sedanolide	C_12_H_18_O_2_	194.13048	195.13776	195.13796	−1.01	28.458	[M + H]
Apigetrin	C_21_H_20_O_10_	432.10558	431.09851	431.09837	0.32	25.431	[M − H]
Nicotiflorin	C_27_H_30_O_15_	594.15817	593.15112	593.15119	−0.12	23.299	[M − H]
Formononetin	C_16_H_12_O_4_	268.07313	269.08035	269.08084	−1.80	24.253	[M + H]
Anisaldehyde	C_8_H_8_O_2_	136.05242	137.05983	137.05971	0.90	11.337	[M + H]
Syringic acid	C_9_H_10_O_5_	198.0524	197.04517	197.04555	−1.91	17.544	[M − H]
Isokaempferide	C_16_H_12_O_6_	300.06318	301.07037	301.07066	−0.98	22.786	[M + H]
3-Coumaric acid	C_9_H_8_O_3_	164.04671	163.03943	163.04007	−3.91	17.081	[M − H]
Vanillic acid	C_8_H_8_O_4_	168.04195	167.03444	167.03498	−3.24	11.371	[M − H]
Rutin	C_27_H_30_O_16_	610.15341	609.14636	609.14611	0.41	24.121	[M − H]
Astilbin	C_21_H_22_O_11_	450.11636	449.10916	449.10893	0.50	21.5	[M − H]
Isovanillic acid	C_8_H_8_O_4_	168.04218	169.04955	169.04954	0.09	5.483	[M + H]
Ferulic acid	C_10_H_10_O_4_	194.05788	195.06517	195.06519	−0.08	20.386	[M + H]
Luteolin	C_15_H_10_O_6_	286.04767	285.04065	285.04046	0.66	25.501	[M − H]
Myricetin	C_15_H_10_O_8_	318.03713	319.0444	319.04484	−1.39	20.833	[M + H]
Sinapinic acid	C_11_H_12_O_5_	224.06839	225.07652	225.07575	3.42	12.824	[M + H]
Neoastilbin	C_21_H_22_O_11_	450.11638	449.1091	449.10893	0.37	16.82	[M − H]
Methyl cinnamate	C_10_H_10_O_2_	162.06793	163.07521	163.07536	−0.90	29.005	[M + H]
Hyperoside	C_21_H_20_O_12_	464.09542	463.08893	463.0882	1.58	22.338	[M − H]
Herbacetin	C_15_H_10_O_7_	302.04277	301.03549	301.03538	0.38	25.629	[M − H]
Methyl gallate	C_8_H_8_O_5_	184.03663	183.0294	183.0299	−2.71	9.638	[M − H]
Isorhamnetin	C_16_H_12_O_7_	316.05763	317.06528	317.06558	−0.94	21.983	[M + H]
Chlorogenic acid	C_16_H_18_O_9_	354.09521	353.08807	353.08781	0.75	11.416	[M − H]
Salicylic acid	C_7_H_6_O_3_	138.03163	139.03896	139.03897	−0.08	11.843	[M − H]
Diosmetin	C_16_H_12_O_6_	300.06316	301.0704	301.07066	−0.88	27.498	[M + H]
Daidzein	C_15_H_10_O_4_	254.05763	255.06491	255.06519	−1.08	24.183	[M + H]
DL-Tryptophan	C_11_H_12_N_2_O_2_	204.08963	203.08235	203.0826	−1.24	7.308	[M − H]
Eugenol	C_10_H_12_O_2_	164.08376	165.09103	165.09101	0.14	23.686	[M + H]
Hydroxygenkwanin	C_16_H_12_O_6_	300.0631	301.07047	301.07066	−0.65	22.614	[M + H]
Umbelliferone	C_9_H_6_O_3_	162.0316	163.03897	163.03897	0.00	11.42	[M + H]
Calycosin	C_16_H_12_O_5_	284.06816	285.07544	285.07575	−1.09	25.519	[M + H]
Medicarpin	C_16_H_14_O_4_	270.08891	271.09619	271.09649	−1.09	26.076	[M + H]
Glabrolide	C_30_H_44_O_4_	468.32353	469.33096	469.33124	−0.59	30.006	[M + H]
Cyanidin	C_15_H_10_O_6_	286.0475	287.05481	287.05501	−0.71	24.619	[M + H]
Cucurbitacin B	C_32_H_46_O_8_	558.31872	603.31769	603.31637	2.18	29.209	[M + COOH-H]

## Data Availability

The raw data supporting the conclusions of this article will be made available by the authors on request.

## Supplementary Materials

The following supporting information can be downloaded at https://www.mdpi.com/article/10.3390/ijms272110018/s1.

## Abbreviations

The following abbreviations are used in this manuscript:

AUC	Area under the curve
DCA	Decision curve analysis
DEG	Differentially expressed gene
FBS	Fetal bovine serum
GLTL	Guilou Tongluo Formula
GSEA	Gene Set Enrichment Analysis
GSVA	Gene Set Variation Analysis
HVGs	Highly Variable Genes
CETSA	Cellular Thermal Shift Assay
DARTS	Drug Affinity Responsive Target Stability
RMSD	Root Mean Square Deviation
RMSF	Root Mean Square Fluctuation
SASA	Solvent Accessible Surface Area
PPI	Protein–Protein Interaction
MCODE	Molecular Complex Detection
OB	Oral Bioavailability
DL	Drug-Likeness
EdU	5-ethynyl-2′-deoxyuridine
PAH	Pulmonary arterial hypertension
PASMC	Pulmonary arterial smooth muscle cell
PCA	Principal component analysis
PDGF	Platelet-derived growth factor
scRNA-seq	Single-Cell RNA sequencing
ssGSEA	Single-Sample Gene Set Enrichment Analysis
WGCNA	Weighted Gene Co-expression Network Analysis
PI3K	Phosphatidylinositol 3-kinase
AKT	Protein kinase B
IGF1R	Insulin-like growth factor 1 receptor
PCNA	Proliferating cell nuclear antigen
IGF1	Insulin-like growth factor 1
PTK2	Protein tyrosine kinase 2
BCL2	B-cell leukemia/lymphoma 2
MD	Molecular dynamics simulations
MCC	Maximal Clique Centrality
MNC	Maximum Neighborhood Component
IC_50_	Half-maximal inhibitory concentration
α-SMA	Alpha-smooth muscle actin
BCA	Bicinchoninic acid
EDTA	Ethylenediaminetetraacetic acid
PVDF	Polyvinylidene difluoride
RIPA	Radioimmunoprecipitation assay
TBST	Tris-buffered saline with Tween 20
PH	Pulmonary hypertension
GEO	Gene Expression Omnibus
UMAP	uniform manifold approximation and projection
DMEM	Dulbecco’s Modified Eagle Medium
ROC	Receiver operating characteristic
KEGG	Kyoto Encyclopedia of Genes and Genomes
GO	Gene Ontology
CCK-8	Cell Counting Kit-8
PFA	Paraformaldehyde
DAPI	4′,6-diamidino-2-phenylindole
BSA	Bovine serum albumin
PBS	Phosphate-buffered saline
