# Supplementary material for: Luteolin and Naringenin from Herbal Medicines Suppress Pulmonary Arterial Smooth Muscle Cell Hyperproliferation in Pulmonary Arterial Hypertension by Modulating IGF1R/PI3K/AKT Signaling
Source: Int J Mol Sci. 2026 Nov 8;27(21):10018. doi: 10.3390/ijms272110018 (PMC13764575; doi:10.3390/ijms272110018)
Supplement: Supplementary file 1 [file ijms-27-10018-s001.zip › Supplementary files.pdf]

Supplementary figures:

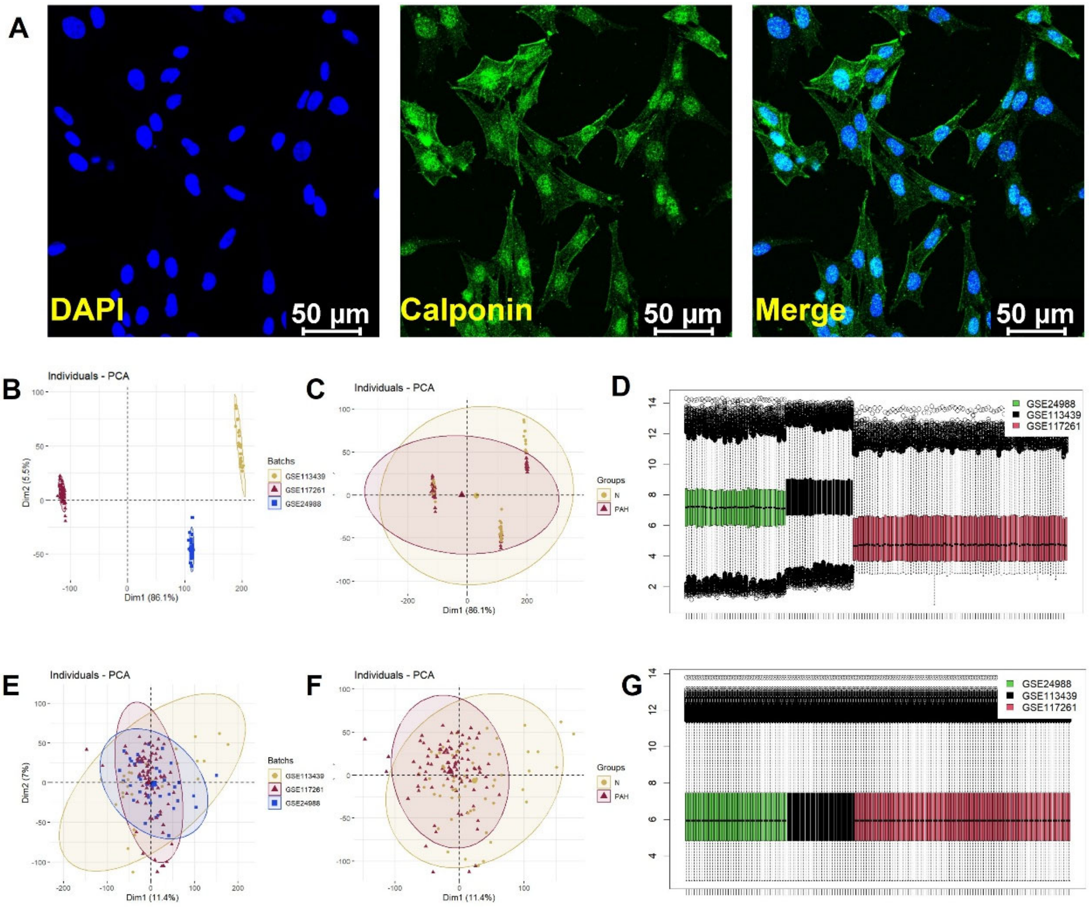

Figure S1. Identification of primary PASMCs and dataset quality control. (A) Identification of PASMC cells by calponin staining. (B) Grouped PCA before batch effect removal. (C) PCA before batch effect removal. (D) Grouped PCA before batch effect removal. (E) Grouped boxplots before batch effect removal. (F) PCA after batch effect removal. (G) Grouped PCA after batch effect removal. (H) Grouped boxplots after batch effect removal. This disease group includes pulmonary arterial hypertension with mixed etiology, labeled as PAH in the figure (see Method 4.3 and Table S4).

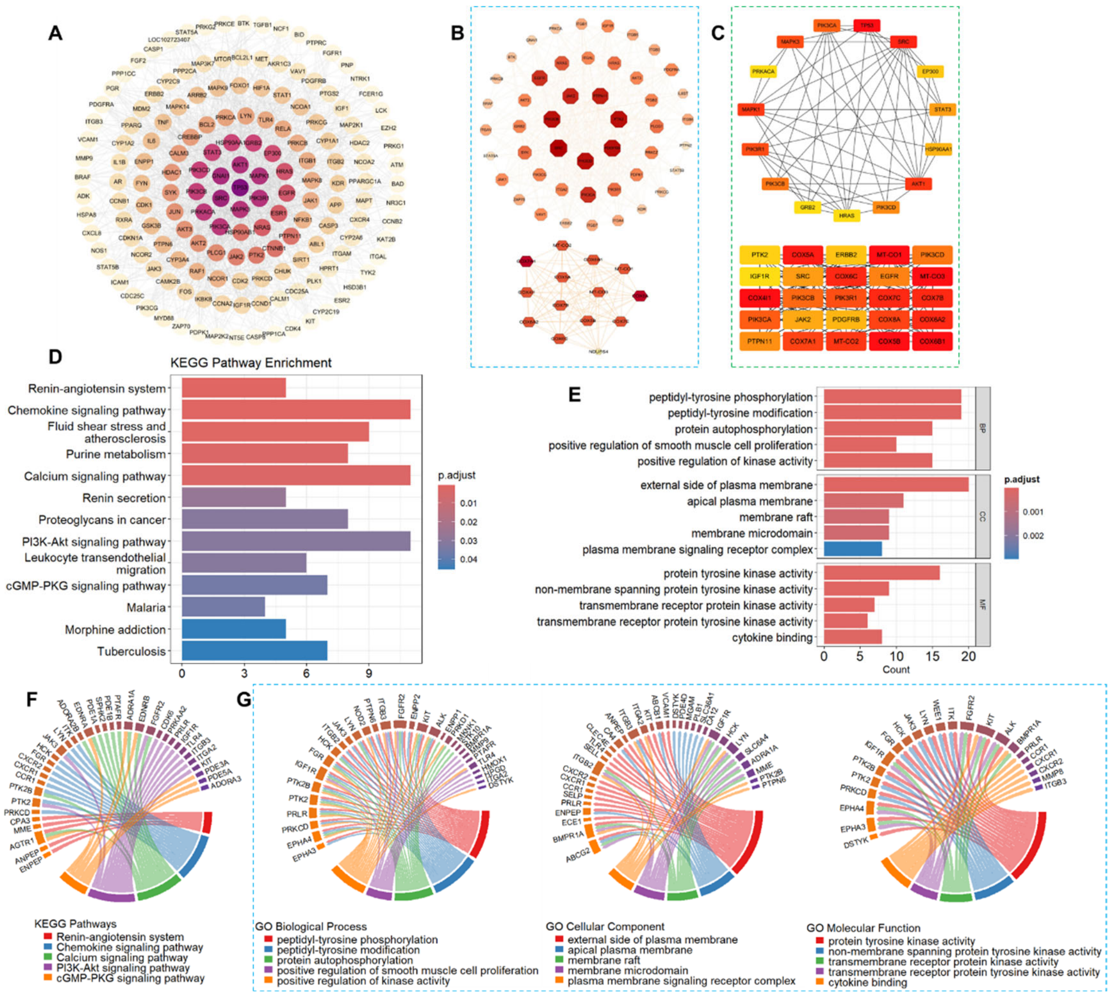

Figure S2. Supplementary data for network pharmacology and enrichment analysis of key gene sets. (A) Target genes screened based on network degree values (values greater than or equal to 2 times the median). (B) Two clusters with high scores were selected based on the MCODE algorithm. (C) Two sets of targets obtained based on MCC and MCC algorithm in Cytohuba. (D) KEGG enrichment bar chart of intersecting genes. (E) GO enrichment analysis bar chart of intersecting genes. (F) KEGG enrichment analysis cnet plot. (G) Cnet plots of GO-BP, GO-CC and GO-MF terms.

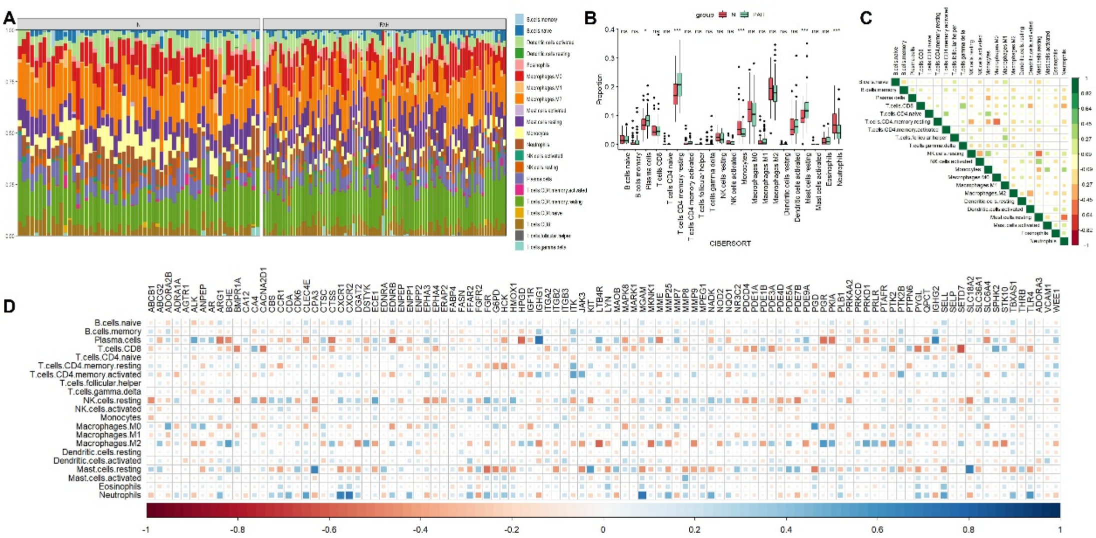

Figure S3. Analysis of lung immune microenvironment reveals pulmonary hypertension-associated immune cell infiltration patterns and key gene set-immune crosstalk networks. (A) Relative abundance and compositional distribution of immune cell subsets in PAH and normal groups. (B) Proportional comparison of differentially infiltrating immune cell subsets between the two groups. (C) Correlation network heatmap of infiltration abundances among immune cell subsets. (D) Association strength heatmap between core gene modules and differential immune cell subsets. *p<0.05, **p<0.01, vs control group. P<0.05 was considered statistically significant. This disease group includes pulmonary arterial hypertension with mixed etiology, labeled as PAH in the figure (see Method 4.3 and Table S4).

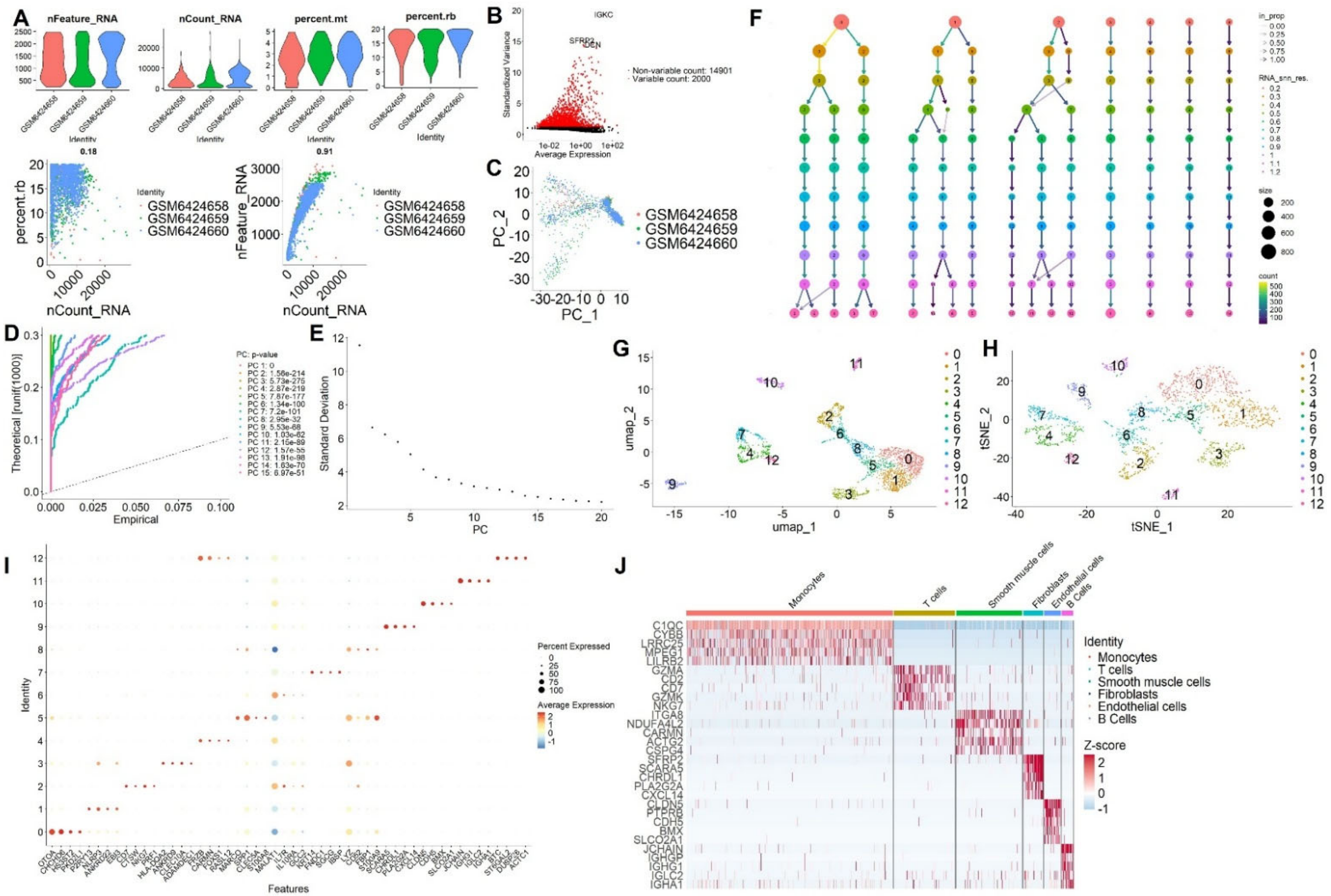

Figure S4. Supplementary materials for dimensionality reduction and annotation of single-cell data. (A) Multi-dimensional quality control metrics assessment of single-cell RNA sequencing data: nFeature-RNA, nCount-RNA, percent.mt, and percent.rb. (B) Scatter plot of expression dispersion distribution for the top 2,000 highly variable genes. (C) Scatter plot of sample distribution following principal component analysis (PCA) dimensionality reduction. (D) and (E) Cumulative variance contribution rates and PC threshold screening. (F) Hierarchical clustering dendrogram of cell samples. (G) and (H) Dimensionality reduction visualization by UMAP and t-SNE dual projection. (I) Bubble plot of highly variable gene expression levels in unannotated cells. (J) Clustering heatmap of highly variable gene expression patterns across annotated cell subpopulations.

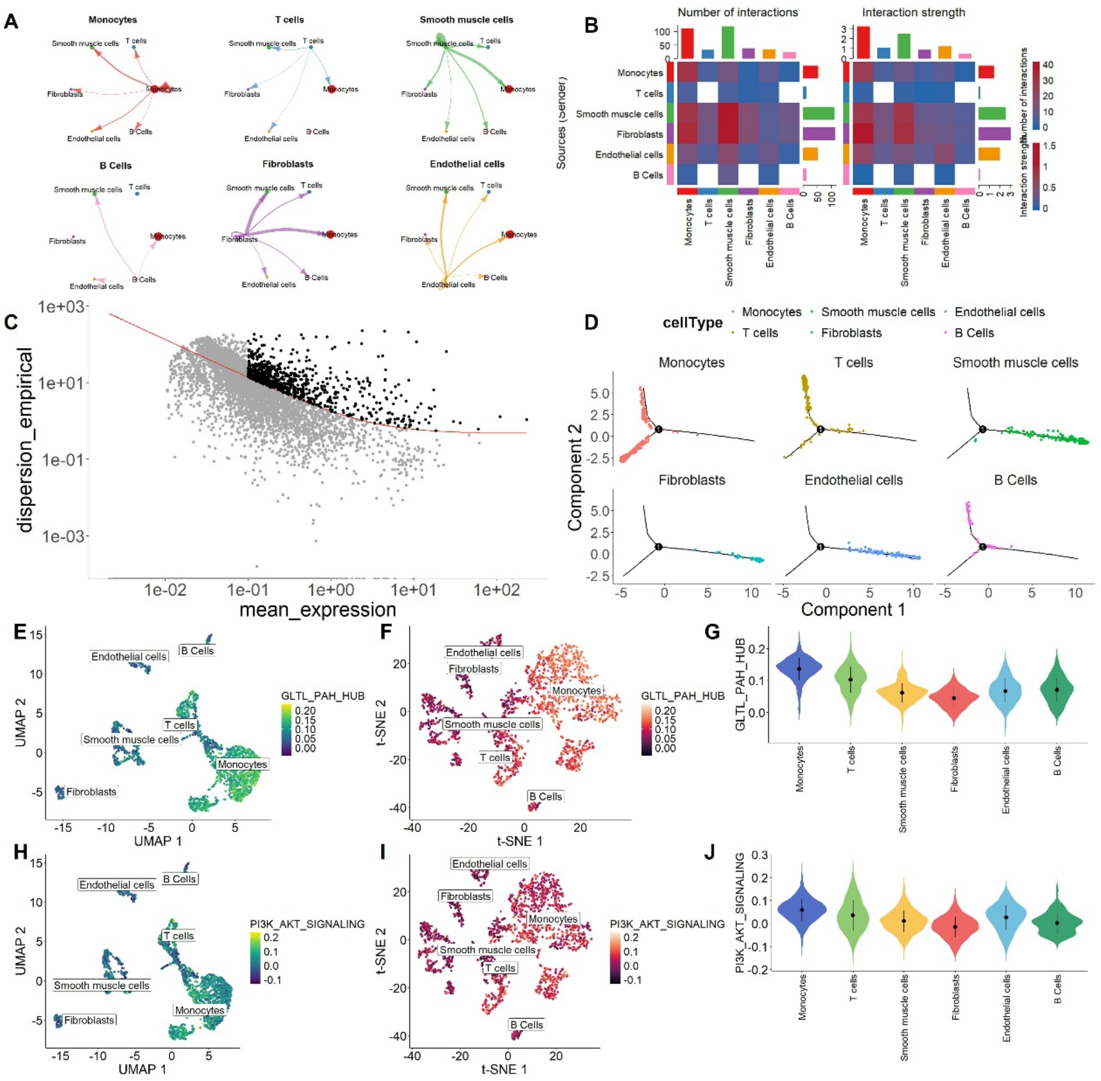

Figure S5. Single-cell transcriptomics reveals cell-cell communication networks, pseudotemporal differentiation trajectories, and signaling pathway activity. (A) and (B) Circular plot (left) and communication intensity heatmap (right) of intercellular ligand-receptor interaction networks. (C) Screening of highly variable genes: scatter plot of genes with average expression > 0.1 and dispersion exceeding the model-fitted value. (D) Construction of pseudotemporal differentiation trajectories and lineage branching patterns across cell subpopulations. (E-G) AddModuleScore activity mapping of the intersection genes: two-dimensional projections by UMAP (E) and t-SNE (F), and violin plots for group comparisons (G). (H-J) AddModuleScore activity mapping of the PI3K-AKT signaling pathway: two-dimensional projections by UMAP (H) and t-SNE (I), with group comparison violin plots (J).

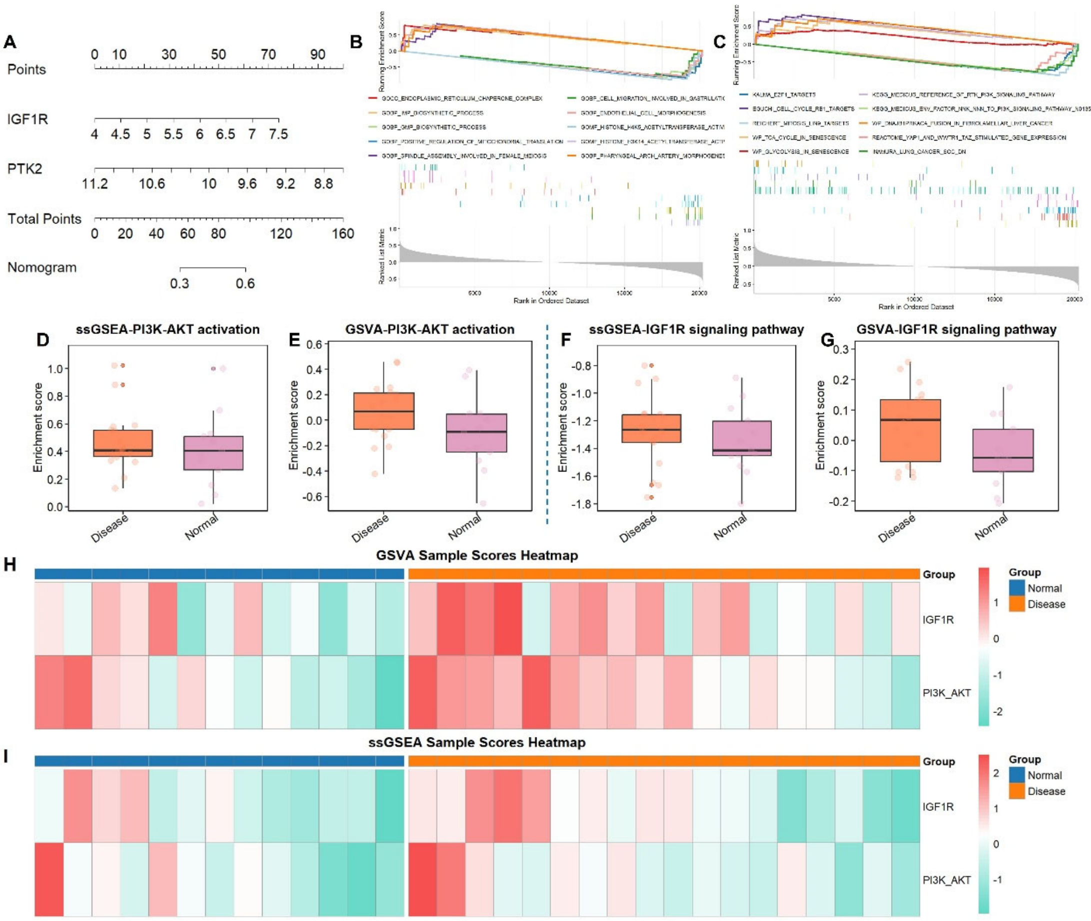

Figure S6. Tissue-level association modelling and enrichment analysis validation of IGF1R and the PI3K/AKT signaling pathway. (A) Nomogram of the multivariable logistic regression model relating candidate target-gene expression to disease status in lung tissue. (B) Significantly enriched biological pathways from GO enrichment analysis of IGF1R single-gene GSEA. (C) Significantly enriched signaling pathways from KEGG enrichment analysis of IGF1R single-gene GSEA. (D) and (E) Boxplot comparisons of ssGSEA (D) and GSVA (E) enrichment scores for the PI3K-AKT signaling pathway in the validation dataset. (F) and (G) Boxplot comparisons of ssGSEA and GSVA enrichment scores for IGF1R signaling pathway in the validation dataset. (H) and (I) Sample-level heatmaps of GSVA (H) and ssGSEA (I) enrichment scores for IGF1R and the PI3K-AKT signaling pathway in the validation dataset.

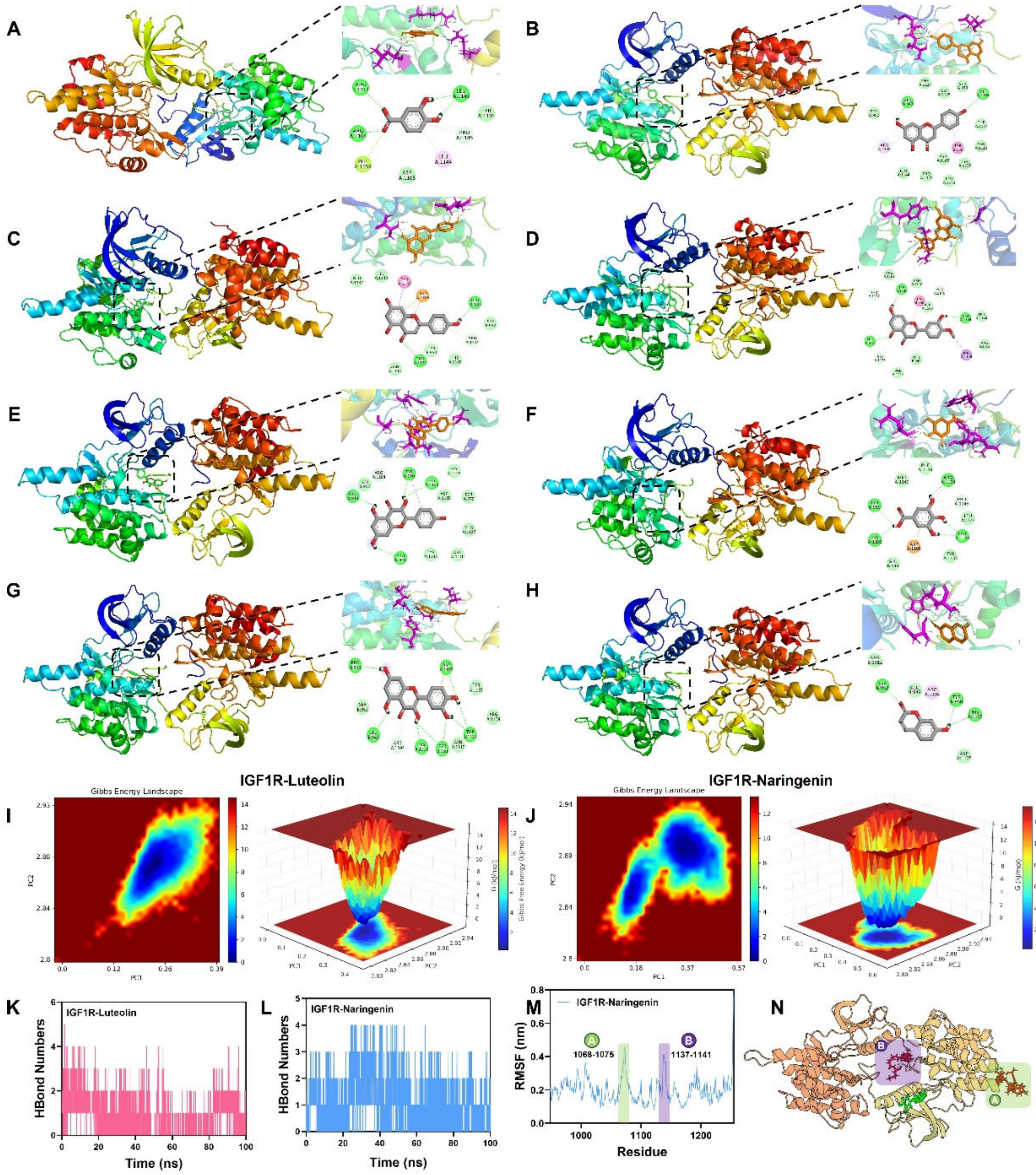

Figure S7. Molecular docking and molecular dynamics simulations predict bioactive components from traditional Chinese medicine that may act on IGF1R. (A) Molecular docking binding mode of protocatechuic acid with IGF1R. (B) Molecular docking binding mode of 5,7-dihydroxy-2-phenylchroman-4-one with IGF1R. (C) Molecular docking binding mode of apigenin with IGF1R. (D) Molecular docking binding mode of diosmetin with IGF1R. (E) Molecular docking binding mode of kaempferol with IGF1R. (F) Molecular docking binding mode of gallic acid with IGF1R. (G) Molecular docking binding mode of quercetin with IGF1R. (H) Molecular docking binding mode of umbelliferone with IGF1R. (I-J) Free energy landscapes of the luteolin (I) and naringenin (J) complexes with IGF1R from molecular dynamics simulations. (K) Heatmap of hydrogen-bond interactions for the luteolin-protein complex. (L) Heatmap of hydrogen-bond interactions for the naringenin-protein complex. (M-N) RMSF-based residue flexibility analysis of the naringenin-protein complex during molecular dynamics simulations.

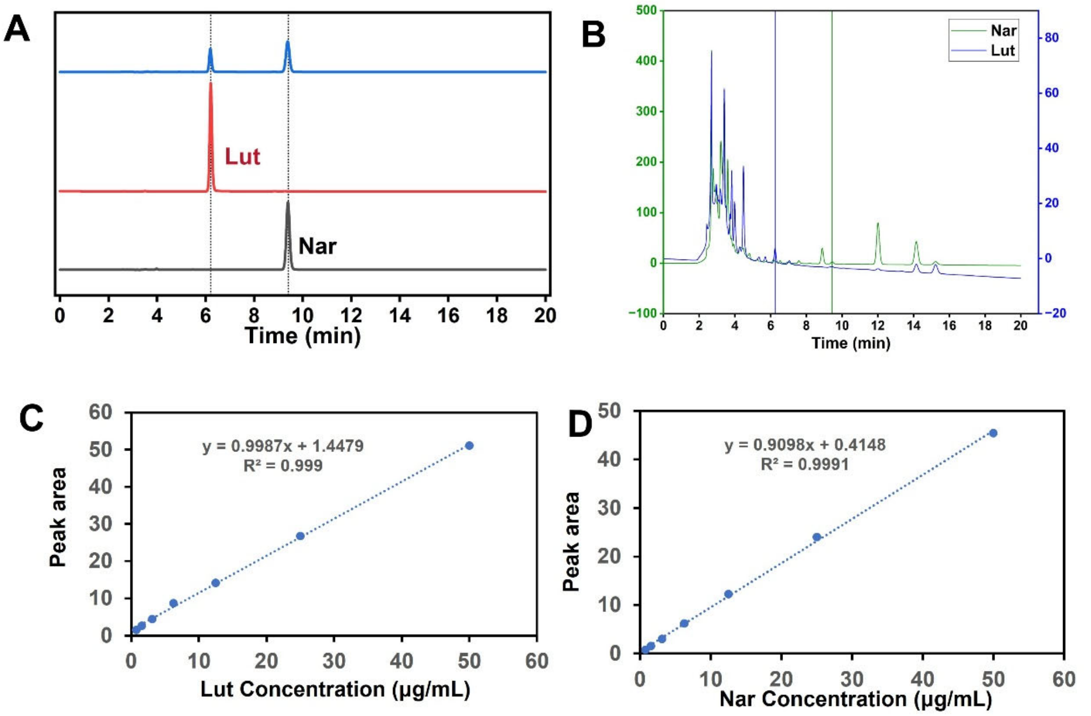

E

Compound	Time (min)	Peak Area	Equation	Concentration (μg/mL)
Nar	9.41	1.886	Y = 0.9098x + 0.4148	1.617
Lut	6.22	2.829	Y = 0.9999x + 1.3876	1.442

F

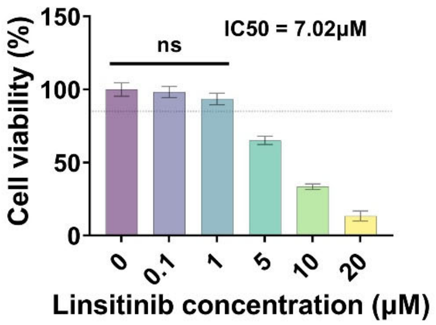

Figure S8. Determination of Luteolin and Naringenin Content in GLTL. (A) Lut and Nar specific attribute detection. (B) GLTL chromatogram with a concentration of 400 μg/mL. (C) Establishment of a Lut calibration curve. (D) Establishment of a Nar calibration curve. (E) Calculation of Lut and Nar content in GLTL. (F) Activity evaluation and IC_{50} value of Linsitinib on PASMCs.

Supplementary tables:

Table S1. The composition of traditional Chinese medicine in GLTL formula

Latin name	Full botanical name	Dosage	Abbreviation
Pericarpium Trichosanthis	*Trichosanthes kirilowii* Maxim.	15g	GL
Herba Ardisiae Japonicae	*Ardisia japonica* (Thunb.) Blume	15g	ADC
Bulbus Allii Macrostemonis	*Allium macrostemon* Bunge	15g	XB
Radix Glycyrrhizae	*Glycyrrhiza uralensis* Fisch.	10g	GC
Ramulus Cinnamomi	*Cinnamomum cassia* (L.) J.Presl	10g	GZ
Herba Ephedrae	*Ephedra sinica* Stapf	10g	MH
Radix Paeoniae Rubra	*Paeonia lactiflora* Pall.	10g	CS
Cortex Moutan	*Paeonia suffruticosa* Andrews	15g	MDP
Poria	*Wolfiporia cocos* (F.A.Wolf) Ryvarden & Gilb. [syn. Poria cocos]	15g	FL
Semen Persicae	*Prunus persica* (L.) Batsch	10g	TR
Radix Platycodonis	*Platycodon grandiflorus* (Jacq.) A.DC.	15g	JG

Table S2. Drug ingredient collection form

Abbreviation	Name	PubChem CID
CS1	3,4-dihydroxybenzoic acid	72
CS2	Hippuric acid	464
CS3	5,7-dihydroxy-2-(4-hydroxyphenyl)chroman-4-one	932
CS4	Syringic acid	10742
CS5	Baicalin	64982
CS6	Phenyl beta-d-glucopyranosiduronic acid	87235
CS7	Paeonimetabolin i	127381
CS8	Campest-5-en-3beta-ol	173183
CS9	(2s,3s,4s,5s,6r)-2-[2-[(5r)-5-ethenyl-5-methyloxolan-2-yl]propan-2-yloxy]-6-[[(2s,3r,4s,5r)-3,4,5-trihydroxyoxan-2-yl]oxymethyl]oxane-3,4,5-triol	190933
CS10	β-sitosterol	222284
CS11	Paeoniflorin	442534
CS12	Stigmast-7-en-3-ol	3080632
CS13	Apigenin	5280443
CS14	Stigmasterol	5280794
CS15	Spinasterol	5281331
CS16	Baicalein	5281605
CS17	Ellagic acid	5281855
CS18	Combretum caffrum	-
CS19	Isosalipurposide	5318659
CS20	Apigenin 7-glucuronide	5319484
CS21	Ethyl oleate	5363269
CS22	3,3'-di-o-methylellagic acid	5488919
CS23	(E)-3-(4-sulfooxyphenyl)prop-2-enoic acid	6070438

CS24	Ferulic acid 4-sulfate	6305574
CS25	Germacrone	6436348
CS26	Protocatechuic acid 3-glucoside	11972438
CS27	3-o-methylellagic acid	13915428
CS28	Lactiflorin	5318917
CS29	Oxypaeoniflora	21631105
CS30	Moudanpioside f	21631108
CS31	Albiflorin	24868421
CS32	4-o-methyldebenzoylpaeoniflorin	44479478
CS33	[(1r,4r)-4-hydroxy-6-methyl-8-oxo-1-[(2s,3r,4s,5s,6r)-3,4,5-trihydroxy-6-(hydroxymethyl)oxan-2-yl]oxy-7-oxatricyclo[4.3.0.03,9]nonan-9-yl]methyl benzoate	51346141
CS34	Paeoniflorigenone	133475
CS35	Protocatechuic acid 4-glucoside	91309592
CS36	(1s,2s,4r)-1,8-epoxy-p-menthan-2-ol glucoside	73815050
CS37	Methylgallic o-sulfate	10825483
CS38	Phenyl hydrogen sulfate	74426
CS39	(+)-Catechin	9064
CS40	Paeoniflorgenone	-
CS41	(1s,2s,4r)-trans-2-hydroxy-1,8-cineole-b-d-glucopyranoside	-
CS42	(2r,3r)-4-methoxyl-distylin	-
CS43	1-o-beta-d-glucopyranosylpaeonisuffrone_qt	-
CS44	Benzoyl paeoniflorin	21631106
CS45	Isobenzoylpaeoniflorin	177827917
CS46	Desbenzoylpaeoniflorin	71452333
MDP1	4-hydroxybenzoic acid	135
MDP2	Citric acid	311

MDP3	Gallic acid	370
MDP4	Succinic acid	1110
MDP5	Vanillin	1183
MDP6	Azelaic acid	2266
MDP7	Dl-3-phenyllactic acid	3848
MDP8	Sucrose	5988
MDP9	(+)-Catechin	9064
MDP10	Paeoniflorin	442534
MDP11	Dihydroxy methoxy acetophenone	13250
MDP12	D-mannose	18950
MDP13	Mairin	64971
MDP14	Pentagalloyl glucose	65238
MDP15	Tetragalloyl glucose	73178
MDP16	3-tert-butyladipic acid	98967
MDP17	Sitosterol	222284
MDP18	Oxypaeoniflora	442534
MDP19	Benzoyloxypaeoniflorin	4628122
MDP20	Quercetin	5280343
MDP21	Kaempferol	5280863
MDP22	[(1r,4r)-4-hydroxy-6-methyl-8-oxo-1-[(2s,3r,4s,5s,6r)-3,4,5-trihydroxy-6-(hydroxymethyl) oxan-2-yl]oxy-7-oxatricyclo [4.3.0.03,9]nonan-9-yl] methyl benzoate	10375422
MDP23	Hyemaloside b	21604164
MDP24	Mudanoside b	21631098
MDP25	Mudanpioside c	21631105
MDP26	Benzoylpaeoniflorin	21631106
MDP27	6'-o-galloyl paeoniflorin	21631107

MDP28	Ethyl gallate	25110931
MDP29	4-o-methylpaeoniflorin	46882921
MDP30	Oxypaeoniflorin	51346141
MDP31	Paeonilactone b	122169317
MDP32	Paeonidanin_qt	-
MDP33	5-[[5-(4-methoxyphenyl)-2-furyl]methylene]barbituric acid	-
MDP34	Mudanpioside-h_qt 2	-
MDP35	Benzoyl paeoniflorin	-
MDP36	Suffruticoside b	-
MDP37	Paeonol	-
GL1	L-Serine	5951
GL2	3,29-Dibenzoyl Rarounitriol	11556558
GL3	guanine	135398634
GL4	L-proline	145742
GL5	β-sitosterol	222284
GL6	L-Glutamic acid	33032
GL7	Linoleic acid	3931
GL8	Naringenin	439246
GL9	Palmitoleic acid	4668
GL10	Quercetin	5280343
GL11	Apigenin	5280443
GL12	Luteolin	5280445
GL13	Isoquercitrin	5280804
GL14	Rutin	5280805
GL15	Kaempferol	5280863
GL16	Cucurbitacin B	5281316

GL17	Cucurbitacin D	5281318
GL18	Diosmetin	5281612
GL19	Kaempferide	5281666
GL20	Thymidine	5789
GL21	L-Alanine	5950
GL22	L-Glutamine	5961
GL23	L-Tyrosine	6057
GL24	L-Leucine	6106
GL25	L-Methionine	6137
GL26	Phenylalanine	6140
GL27	L-Threonin	6288
GL28	L-Tryptophan	6305
GL29	L-Arginine	6322
GL30	Tangeretin	68077
GL31	α-Linolenic acid	860
GL32	5-dehydrokarounidiol	10094697
GL33	10α-cucurbita-5,24-diene-3β-ol	-
GL34	karounidiol 3-o-benzoate	-
GL35	7-oxo-dihydrokaro-unidiol	-
FL1	3,4-dihydroxybenzoic acid	72
FL2	Adenine	190
FL3	Citric acid	311
FL4	Succinic acid	1110
FL5	Vanillin	1183
FL6	Azelaic acid	2266
FL7	Sucrose	5988

FL8	D-mannose	18950
FL9	Hederagenin	73299
FL10	Eburicoic acid	73402
FL11	3-tert-butyladipic acid	98967
FL12	Poricoic acid b	3086648
FL13	Ergosta-7,22e-dien-3beta-ol	5283628
FL14	Ergosterol peroxide	5351516
FL15	Poricoic acid a	5471851
FL16	Poricoic acid b	5471852
FL17	Poricoic acid g	5471966
FL18	Pachymic acid	5484385
FL19	Acetylursolic acid	6475119
FL20	Polyporenic acid c	9805290
FL21	3-o-acetyl-16alpha-hydroxytrametenolic acid	9958136
FL22	3-epidehydrotumulosic acid	10005581
FL23	Cerevisterol	10181133
FL24	16alpha-hydroxydehydrotrametenolic acid	10743008
FL25	Poricoic acid h	10918099
FL26	Dimethyl d-malate	11062697
FL27	Daedaleanic acid a	11179164
FL28	Fomefficinic acid a	11431307
FL29	Daedaleanic acid b	11465951
FL30	Trametenolic acid	12309443
FL31	Tumulosic acid	12314446
FL32	Dehydrotumulosic acid	15225964
FL33	Poricoic acid e	15225966

FL34	3-epidehydropachymic acid	15226716
FL35	Dehydropachymic acid	15226717
FL36	Dehydroeburicoic acid	15250826
FL37	3-dehydrotrametenolic acid	15391340
FL38	16-deoxyporicoic acid b	16736458
FL39	25-hydroxyporicoic acid h	16736865
FL40	25-hydroxyporicoic acid c	44556811
FL41	6,7-dehydroporicoic acid h	44556878
FL42	Poricoic acid am	46882717
FL43	Poricoic acid c	56668247
FL44	Poricoic acid ce	102480393
FL45	29-hydroxydehydropachymic acid	137355885
FL46	(2r)-2-[(3s,5r,10s,13r,14r,16r,17r)-3,16-dihydroxy-4,4,10,13,14-pentamethyl-2,3,5,6,12,15,16,17-octahydro-1h-cyclopenta[a]phenanthren-17-yl]-6-methyl-5-oxoheptanoic acid	137648664
FL47	(2r)-2-[(3r,5r,10s,13r,14r,16r,17r)-3,16-dihydroxy-4,4,10,13,14-pentamethyl-2,3,5,6,12,15,16,17-octahydro-1h-cyclopenta[a]phenanthren-17-yl]-6-methyl-5-oxoheptanoic acid	137654023
FL48	7,9(11)-dehydropachymic acid	-
FL49	3β-hydroxy-16α-acetoxy-lanosta-7,9(11),24-trien-21-oic acid	-
FL50	6-hydroxy-dehydrotumulosic acid	-
FL51	25-hydroxypachymic acid	-
FL52	Pachymic acid c	-
ADC1	Cianidanol	9064
ADC2	4-heptylbenzoic acid	170036
ADC3	Rapanone	100659
ADC4	Carvacrol	10364
ADC5	Oleanolic acid	10494

ADC6	Epicatechin gallate	107905
ADC7	Iso-e super	108242
ADC8	Astilbin	119258
ADC9	Perillic acid	1256
ADC10	Nootkatone	1268142
ADC11	Tetradecanedioic acid	13185
ADC12	4-oxododecanedioic acid	13213508
ADC13	Ethyl gallate	13250
ADC14	Luteolin-7-o-rutinoside	14032966
ADC15	9s,13r-12-oxophytodienoic acid	14037063
ADC16	Vitamin-e	14985
ADC17	Thymoxyacetic acid	220116
ADC18	3-oxocyclopentanecarboxylic acid	227798
ADC19	3-hydroxycholest-5-en-7-one	287664
ADC20	1-salicylate glucuronide	3084023
ADC21	Citric acid	311
ADC22	Embelin	3218
ADC23	Salicylic acid	338
ADC24	Gentisic acid	3469
ADC25	Hexylresorcinol	3610
ADC26	Gallic acid	370
ADC27	Methyl salicylate	4133
ADC28	Myristicin	4276
ADC29	Taxifolin	439533
ADC30	Eriodictyol	440735
ADC31	Schottenol	441837

ADC32	Docosahexaenoic acid	445580
ADC33	Palmitoleic acid	445638
ADC34	Mycophenolic acid	446541
ADC35	(2e)-3-(2,3-dihydro-1,4-benzodioxin-6-yl)acrylic acid	4644031
ADC36	Phenylbutyric acid	4775
ADC37	Fraxetin	5273569
ADC38	Quercetin	5280343
ADC39	Apigenin	5280443
ADC40	Luteolin	5280445
ADC41	Scopoletin	5280460
ADC42	Cynaroside	5280637
ADC43	Apigenin-7-o-glucoside	5280704
ADC44	Rutin	5280805
ADC45	Kaempferol	5280863
ADC46	(±)-Abscisic acid	5280896
ADC47	Spinasterol	5281331
ADC48	Umbelliferone	5281426
ADC49	Diosmetin	5281612
ADC50	Hyperoside	5281643
ADC51	Myricetin	5281672
ADC52	Myricitrin	5281673
ADC53	Mandenol	5282184
ADC54	Afzelin	5316673
ADC55	Hydroxygenkwanin	5318214
ADC56	Linolenic acid ethyl ester	5367460

ADC57	Docosapentaenoic acid	5497182
ADC58	Hydroxyprogesterone	6238
ADC59	Methyl cinnamate	637520
ADC60	Epigallocatechin gallate	65064
ADC61	Bergenin	66065
ADC62	Caffeic acid	689043
ADC63	4-methoxycinnamic acid	699414
ADC64	3,4-dimethoxybenzoic acid	7121
ADC65	Epigallocatechin	72277
ADC66	4-tert butylbenzoic acid	7403
ADC67	Methyl gallate	7428
ADC68	Dl-carvone	7439
ADC69	P-cymene	7463
ADC70	Monobutyl phthalate	8575
ADC71	Apigenin-7-o-rutinoside	9851181
ADC72	3-tert-butyladipic acid	98967
ADC73	Ardisinol i	5319846
ADC74	Ardisinol ii	6454482
XB1	Macrostemonoside f	192523
XB2	Macrostemonoside a	197480
XB3	Macrostemonoside d	197481
XB4	Macrostemonoside e	3083447
XB5	Quercetin	5280343
XB6	Prostaglandin b1	5280388
XB7	Prostaglandin a1	5281912
XB8	Sitosteryl acetate	5354503

XB9	N-cis-feruloyltyramine	6440659
XB10	Beta-sitosterol	222284
XB11	Paprazine	5372945
XB12	Sitostanol-5,6,22,23-d4	71309302
XB13	Hesperetin	72281
XB14	Naringenin	932
XB15	(Z)-3-(4-hydroxy-3-methoxy-phenyl)-n-[2-(4-hydroxyphenyl)ethyl]acrylamide	-
XB16	Macrostemonoside e_qt	-
GC1	(2r)-7-hydroxy-2-(4-hydroxyphenyl)chroman-4-one	928837
GC2	Prunin	92794
GC3	Vestitol	92503
GC4	Inermine	91510
GC5	Licoricesaponin e2	86278258
GC6	Liquiritigenin-7,4'-diglucoside	73981649
GC7	Sigmoidin-b	73205
GC8	M49	71598534
GC9	Formononetin 7-o-glucuronide	71316927
GC10	Quinic acid	6508
GC11	Mairin	64971
GC12	2-(3,4-dihydroxyphenyl)-5,7-dihydroxy-6-(3-methylbut-2-enyl)chromone	6450964
GC13	Isoliquiritin apioside	6442433
GC14	Isoliquiritigenin	638278
GC15	(2s)-6-(2,4-dihydroxyphenyl)-2-(2-hydroxypropan-2-yl)-4-methoxy-2,3-dihydrofuro[3,2-g]chromen-7-one	637112
GC16	Licoagroisoflavone	636883
GC17	Sophoraflavone b	5491513

GC18	Gancaonin h	5481949
GC19	Semilicoisoflavone b	5481948
GC20	Licoisoflavone b	5481234
GC21	Gadelaidic acid	5460988
GC22	Pallidiflorin	5320382
GC23	Neoisoliquiritin	5320092
GC24	Glycyrol	5320083
GC25	3'-methoxyglabridin	5319439
GC26	Licoricone	5319013
GC27	Licochalcone b	5318999
GC28	Licochalcone a	5318998
GC29	Jaranol	5318869
GC30	Isotrifoliol	5318679
GC31	Isoliquiritin	5318591
GC32	Isolicoflavonol	5318585
GC33	Inflacoumarin a	5318437
GC34	Glyzaglabrin	5317777
GC35	Glypallichalcone	5317768
GC36	Glycyrrhiza flavonol a	5317765
GC37	Glycycoumarin	5317756
GC38	Glabrone	5317652
GC39	Lupiwighteone	5317480
GC40	Gancaonin b	5317479
GC41	Gancaonin a	5317478
GC42	Eurycarpin a	5317300
GC43	Icos-5-enoic acid	5312522

GC44	7,4'-dihydroxyflavone	5282073
GC45	Licoisoflavone	5281789
GC46	Isorhamnetin	5281654
GC47	Glepidotin a	5281619
GC48	Umbelliferone	5281426
GC49	Kaempferol	5280863
GC50	Chrysoeriol	5280666
GC51	Formononetin	5280378
GC52	Quercetin	5280343
GC53	Neoliquiritin	51666248
GC54	Liquiritin	503737
GC55	Licocoumarone	503731
GC56	Licochalcone g	49856081
GC57	Isovitexin 2"-o-beta-d-glucoside(1-)	49791997
GC58	1-methoxyphaseollidin	480873
GC59	Glyasperin c	480859
GC60	Glycyrin	480787
GC61	Glyasperin b	480784
GC62	Gancaonin g	480780
GC63	Dehydroglyasperin c	480775
GC64	Glabrene	480774
GC65	Licoricesaponin c2	452864
GC66	Trifolirhizin	442827
GC67	Ononin	442813
GC68	Violanthin	442665
GC69	Vicenin-2	442664

GC70	Schaftoside	442658
GC71	Phaseol	44257530
GC72	Glepidotin b	442411
GC73	Naringenin	439246
GC74	Licorice glycoside e	42607811
GC75	Licoisoflavanone	392443
GC76	Glyasperin f	392442
GC77	Isoformononetin	3764
GC78	Medicarpin	336327
GC79	7-acetoxy-2-methylisoflavone	268208
GC80	7,2',4'-trihydroxy − 5-methoxy-3 − arylcoumarin	25015742
GC81	(-)-Medicocarpin	23724664
GC82	Tachiogroside b	22297602
GC83	Sitosterol	222284
GC84	(2s)-2-[4-hydroxy-3-(3-methylbut-2-enyl)phenyl]-8,8-dimethyl-2,3-dihydropyrano[2,3-f]chromen-4-one	197678
GC85	3,22-dihydroxy-11-oxo-delta(12)-oleanene-27-alpha-methoxycarbonyl-29-oic acid	195396
GC86	Glabrolide b	163304465
GC87	Phaseolinisoflavan	162412
GC88	Licoagrocarpin	15840593
GC89	Isoglabrolide	15559941
GC90	Kanzonol w	15380912
GC91	Glycyrrhizic acid	14982
GC92	Licoricesaponin g2	14891565
GC93	Xambioona	14769500
GC94	Monoglucuronylglycyrrhetinic acid	14312747

GC95	Licuraside	14282455
GC96	Licoricesaponin a3	14187172
GC97	Odoratin	13965473
GC98	Licoricesaponin b2	13457500
GC99	6''-acetylliquiritin	131753130
GC100	Licoricesaponin h2	12889143
GC101	Glabridin	124052
GC102	Isoglycyrol	124050
GC103	Glabranin	124049
GC104	Licopyranocoumarin	122851
GC105	Liquiritigenin	114829
GC106	(E)-3-[3,4-dihydroxy-5-(3-methylbut-2-enyl)phenyl]-1-(2,4-dihydroxyphenyl)prop-2-en-1-one	11267805
GC107	(E)-1-(2,4-dihydroxyphenyl)-3-(2,2-dimethylchromen-6-yl)prop-2-en-1-one	10881804
GC108	Oleanolic acid	10494
GC109	3'-hydroxy-4'-o-methylglabridin	10338211
GC110	Methyl-liquiritigenin	10265122
GC111	[4-[(s)-4-oxo-7-hydroxychroman-2-yl]phenyl]beta-d-glucopyranosiduronic acid	102065875
GC112	Glycyroside	101939210
GC113	Licorice glycoside b	101938904
GC114	Licorice glycoside a	101938903
GC115	Quillaic acid	101810
GC116	Kanzonol f	101666840
GC117	Glyasperin m	101664572
GC118	Licoricesaponin k2	101589724
GC119	Formononetin 7-glucuronide	101220281
GC120	Enoxolone	10114

GC121	Glucoliquiritin apioside	10055615
GC122	Topazolin	5481965
GC123	Quercetin der.	5316900
GC124	Calycosin	5280448
GC125	Helichrysin a	15559669
GC126	Shinpterocarpin	10336244
GC127	Liquiritin apioside	10076238
GC128	8-prenylated eriodictyol	-
GC129	7-methoxy-2-methyl isoflavone	-
GC130	18α-hydroxyglycyrrhetic acid	-
GC131	6-prenylated eriodictyol	-
GC132	Euchrenone	-
TR1	Tryptophan	6305
TR2	5,7-Dihydroxy-2-phenylchroman-4-one	68071
TR3	Palmitic acid	985
TR4	Stearic acid	5281
TR5	Heptanoic acid	8094
TR6	Campesterol	173183
TR7	Oleic acid	445639
TR8	Amygdalin	656516
TR9	Linoleic acid	5280450
TR10	Kaempferol	5280863
TR11	9-hexadecenoic acid	5282745
TR12	Ga120	100947720
TR13	Ga119	100947721
TR14	Ga87	101663385

TR15	Ga54	12310181
TR16	Ga60	13071237
TR17	Ga77	131752128
TR18	Ga63	14160552
TR19	Ga30	14833709
TR20	Beta-sitosterol	222284
TR21	Gibberellin a19	5460209
TR22	Gibberellin a44	5460372
TR23	Gibberellin 17	5460657
TR24	3-o-p-coumaroylquinic acid	6441280
TR25	Hederagenin	73299
TR26	Gibberellin 7	92782
TR27	Sitosterol alpha1	9548595
TR28	2,3-didehydro ga70	-
TR29	2,3-didehydro ga77	-
TR30	Ga121-isolactone	-
TR31	Populoside_qt	
MH1	Ephedrine	9294
MH2	(+)-Catechin	9064
MH3	Delphinidin	68245
MH4	Truflex obp	66540
MH5	Supraene	638072
MH6	Pectolinarigenin	5320438
MH7	Mandenol	5282184
MH8	Genkwanin	5281617
MH9	Diosmetin	5281612

MH10	Kaempferol	5280863
MH11	Stigmasterol	5280794
MH12	Herbacetin	5280544
MH13	Luteolin	5280445
MH14	Quercetin	5280343
MH15	Poriferast-5-en-3beta-ol	457801
MH16	Resivit	445881
MH17	Eriodictyol	440735
MH18	Taxifolin	439533
MH19	Naringenin	439246
MH20	Leucopelargonidin	3286789
MH21	Beta-sitosterol	222284
MH22	Campest-5-en-3beta-ol	173183
MH23	24-ethylcholest-4-en-3-one	15596633
MH24	(+)-Leucocyanidin	155206
MH25	Caffeic acid glucuronidation	25171992
MH26	Cathine	441457
MH27	Norephedrine	26934
MH28	Pseudoephedrine	7028
MH29	N-methylephedrine	4374
JG1	Linolenic acid	5280934
JG2	Robinin	5281693
JG3	Spinasterol	5281331
JG4	Luteolin	5280445
JG5	Acacetin	5280442
JG6	Cis-dihydroquercetin	443758

JG7	2-o-methyl-3—o-β-d-glucopyranosyl platycogenate a	-
JG8	Dimethyl 2-o-methyl-3-o-a-d-glucopyranosyl platycogenate a	-
JG9	Platycodin d	162859
JG10	Platycodin d3	75251137
JG11	Platicodigenin	12314399
JG12	Platycodin d2	53317652
JG13	3-o-beta-d-glucopyranosylplatycodigenin	102185205
JG14	Platycoside k	102004765
JG15	Platycoside f	101048500
JG16	Deapio platycodin d3	70698190
JG17	Lobetyolin	53486204
JG18	Platycoside l	11556931
JG19	Platycoside a	50900942
JG20	Platyconic acid c	102052424
JG21	Polygalacin d	75144769
JG22	Platycodon a	102189085
JG23	A-spinasterol	5281331
JG24	Platycogenic acid c	69569686
JG25	Apigenin	5280443
JG26	Platycogenin a	78100583
GZ1	Cinnamic acid	444539
GZ2	3,4-dihydroxybenzoic acid	72
GZ3	Coumarin	323
GZ4	Gallic acid	370
GZ5	Octanal	454
GZ6	Hippuric acid	464

GZ7	Pseudoephedrine	7028
GZ8	6-methylcoumarin	7092
GZ9	Ephedrine	9294
GZ10	7-methylcoumarin	17131
GZ11	Cinnamaldehyde	637511
GZ12	2-hydroxycinnamic acid	637540
GZ13	3-hydroxycinnamic acid	637541
GZ14	4-hydroxycinnamic acid	637542
GZ15	(-)-Taxifolin	712316
GZ16	Cinnamyl alcohol	5315892
GZ17	Sydowic acid	11737144
GZ18	Protocatechuic acid 3-glucoside	11972438
GZ19	Isovitexin 2''-o-beta-d-glucoside(1-)	49791997
GZ20	Ent-epicatechin	182232
GZ21	Beta-sitosterol	222284
GZ22	Taxifolin	439533
GZ23	Peroxyergosterol	5351516
GZ24	(+)-Catechin	9064
GZ25	Epicatechin	72276
GZ26	Perseanol	44559454
GZ27	Quercetin	5280343
GZ28	Truxinic acid	266036

Table S3. Molecular Docking Results Table

Compounds	Binding energy(kcal/mol)	Related residues	Intermol energy	Number of hydrogen bonds
Protocatechuic acid	-3.79	LEU-1143, ARG-1104, ARG-1012	-4.98	3
Pinocembrin (5,7-dihydroxy-2-phenylchroman-4-one)	-4.68	MET-1126, THR-1127, GLU-949	-5.88	3
Apigenin	-4.79	THR-1127, GLU-949	-5.98	2
Diosmetin	-4.68	TYR-1135, ARG-1104, GLY-1125	-6.17	2
Kaempferol	-4.8	HIS-1103, SER-952, GLU-949, TYR-950, THR-1127	-6.29	3
Gallic acid	-3.85	LEU-1143, ARG-1104, TYR-950, HIS-1103	-5.34	2
Luteolin	-5.39	ARG-1104, GLU-949, GLY-1125	-6.88	4
Quercetin	-4.98	THR-1127, ASN-1110, TYR-1135, HIS-1103, GLU-949	-6.77	2
Umbelliferone	-4.3	HIS-1103, SER-952, TYR-950	-4.6	2
Naringenin	-5.04	LEU-1143, PRO-948	-6.24	2

Table S4. Data Source Table

GEO accession	Platform	Organism	Experiment type	Status	Samples (PH/N)
GSE24988	GPL6244	Homo sapiens	Expression profiling by array	Public on Jun 01, 2011	17/22
GSE113439	GPL6244	Homo sapiens	Expression profiling by array	Public on Apr 21, 2018	15/11
GSE117261	GPL6244	Homo sapiens	Expression profiling by array	Public on Dec 23, 2018	58/25
GSE15197	GPL6480	Homo sapiens	Expression profiling by array	Public on Jan 22, 2010	18/13
GSE228644	GPL20301	Homo sapiens	Expression profiling by high throughput sequencing	Public on Mar 31, 2023	3/0

GSE24988 comprises lung tissue samples from 116 patients with pulmonary fibrosis (PF). Pulmonary artery pressures were recorded intraoperatively via right heart catheterization. Patients were stratified into four groups based on mean pulmonary arterial pressure (mPAP): No PH (mPAP < 20 mmHg, n = 22), Intermediate PH (mPAP 21–39 mmHg, n = 45), Severe PH (mPAP > 40 mmHg, n = 17), and a Validation Set (n = 32). This study used the severe pulmonary artery pressure group. This dataset therefore represents Group 3 PH (PH associated with lung disease and/or hypoxia), specifically PH secondary to PF, rather than Group 1 PAH.

Table S4b. Additional pulmonary arterial hypertension datasets surveyed in this study (Group 1 PAH / IPAH). These datasets are listed for completeness and were not merged into the bulk training sets described in Table S4.

GEO accession	Platform	Organism	Experiment type	Samples
GSE22356	GPL570	Homo sapiens	Expression profiling by array	IPAH (8)/N (10)
GSE69416	GPL17077	Homo sapiens	Expression profiling by array	PAH (6)/N (6)
GSE131793	GPL6244	Homo sapiens	Expression profiling by array	PAH (10)/N (10)
GSE38267	GPL13607	Homo sapiens	Expression profiling by array	PAH (13)/N (28)
GSE256539	GPL30991	Homo sapiens	Expression profiling by array	PAH (149)/N (62)
GSE73674	GPL20982	Homo sapiens	Expression profiling by array	PAH (6)/N (9)
GSE33463	GPL6947	Homo sapiens	Expression profiling by array	IPAH (30)/N (41)

Table S5. Liquid chromatography conditions

Time (min)	Phase A (%)	Phase B (%)
0	98	2
15	90	10
20	80	20
25	75	25
30	55	45
35	5	95
38	0	100

Instrument model: Q Exactive™ Quadrupole-Orbitrap™ High resolution mass spectrometer (Thermo Fisher Scientific, USA)

Chromatography column: Thermo Scientific Accucore™ C18 (3 mm × 100 mm, 2.6 µm)

Mobile phase: A: 0.1% formic acid aqueous solution; B: Acetonitrile; Flow rate: 0.5 mL/min; column temperature: 40℃; injection volume: 5 µL.

The content of luteolin and naringenin in the freeze-dried GLTL extract was determined using high-performance liquid chromatography (HPLC) with external calibration. The chromatographic analysis was performed on a Thermo Ultimate 3000 HPLC system equipped with a diode array detector, using a Hypersil GOLD C18 column (250 mm × 4.6 mm, 5 µm; Thermo Fisher Scientific). The mobile phase consisted of acetonitrile–0.1% formic acid aqueous solution (35:65, v/v), eluted in isocratic mode at a flow rate of 1.0 mL/min; the column temperature was maintained at 30 °C, the injection volume was 10 µL, and the detection wavelengths were

set to 288 nm and 350 nm, respectively. A standard curve was plotted using luteolin and naringenin reference standards. A 1.0 mg/mL stock solution was first prepared in methanol, then serially diluted with methanol to seven concentration levels (50, 25, 12.5, 6.25 μg/mL, and so on); the standard curve was plotted via linear regression analysis of peak areas versus concentration. Subsequently, a 400 μg/mL sample of the GLTL extract was injected to determine the content of luteolin and naringenin.

Table S6. Mass spectrometry conditions

Parameters	Value
Scanning mode	Full MS/dd-MS^2
Ion source	ESI
Full MS resolution	70,000
MS/MS resolution	17,500
Scanning range	75-1100 m/z
Collision energy	20, 40, 60 eV
Spray voltage	Positive mode 3.5 kV, negative mode 3.0 kV
Sheath gas velocity	40 arb
Auxiliary gas flow rate	15 arb
Capillary temperature	350°C
Auxiliary gas heating temperature	300°C

Table S7. Primer sequences used in qPCR.

Gene Name	Forward (5′→3′)	Reverse (5′→3′)
PCNA	TCACGTCTCCTTAGTGCAGC	GGAGACAGTGGAGTGGCTTT
CCND1	CTGGATGCTAGAGGTCTGCG	CAGGCTTGACTCCAGAAGGG
BCL2	TGGCTGGTGTAGTTCTGCTG	TAATAGGGGAAGGGGCGTGA
IGF1	TGAGCGCACCTCCAATAAAGA	CCTTCTCCTTTGCAGCTTCC
IGF1R	AGTCCTGATTCCTCCGAGCT	ACTGGGCTCCATCTCATCCT
GAPDH	GGCAAGTTCAACGGCACAG	CGCCAGTAGACTCCACGACAT
